# The Potential of Single-Cell Oils Derived From Filamentous Fungi as Alternative Feedstock Sources for Biodiesel Production

**DOI:** 10.3389/fmicb.2021.637381

**Published:** 2021-01-28

**Authors:** Sizwe I. Mhlongo, Obinna T. Ezeokoli, Ashira Roopnarain, Busiswa Ndaba, Patrick T. Sekoai, Olivier Habimana, Carolina H. Pohl

**Affiliations:** ^1^Discipline of Medical Microbiology, School of Laboratory Medicine and Medical Sciences, Medical School, University of KwaZulu-Natal, Durban, South Africa; ^2^Department of Microbial, Biochemical and Food Biotechnology, University of the Free State, Bloemfontein, South Africa; ^3^Microbiology and Environmental Biotechnology Research Group, Institute for Soil, Climate and Water, Agricultural Research Council, Pretoria, South Africa; ^4^The School of Biological Sciences, The University of Hong Kong, Pokfulam, Hong Kong

**Keywords:** single-cell oils, filamentous fungi, biorefinery, feedstock, biodiesel

## Abstract

Microbial lipids, also known as single-cell oils (SCOs), are highly attractive feedstocks for biodiesel production due to their fast production rates, minimal labor requirements, independence from seasonal and climatic changes, and ease of scale-up for industrial processing. Among the SCO producers, the less explored filamentous fungi (molds) exhibit desirable features such as a repertoire of hydrolyzing enzymes and a unique pellet morphology that facilitates downstream harvesting. Although several oleaginous filamentous fungi have been identified and explored for SCO production, high production costs and technical difficulties still make the process less attractive compared to conventional lipid sources for biodiesel production. This review aims to highlight the ability of filamentous fungi to hydrolyze various organic wastes for SCO production and explore current strategies to enhance the efficiency and cost-effectiveness of the SCO production and recovery process. The review also highlights the mechanisms and components governing lipogenic pathways, which can inform the rational designs of processing conditions and metabolic engineering efforts for increasing the quality and accumulation of lipids in filamentous fungi. Furthermore, we describe other process integration strategies such as the co-production with hydrogen using advanced fermentation processes as a step toward a biorefinery process. These innovative approaches allow for integrating upstream and downstream processing units, thus resulting in an efficient and cost-effective method of simultaneous SCO production and utilization for biodiesel production.

## Introduction

Biofuels derived from low-cost substrates such as agro-industrial waste are promising alternative sources of liquid energy (Carvalho et al., 2015; Dong et al., 2016). Bio-based fuel can enable energy independence, reduce greenhouse gas emissions, and enhance sustainable economic development (Xia et al., 2011). Unlike fossil-based fuels, which negatively impacts the environment, biodiesel is considered a clean, renewable, and sustainable liquid fuel, which can replace fossil-based diesel (de Jong et al., 2012; Chopra et al., 2016). Biodiesel’s advantages include high energy density, lubricity, fast biodegradation rate, and low greenhouse gas emissions (Vicente et al., 2010). However, the current production of biodiesel relies on conventional sources such as plants and animals, which results in high feedstock costs, competition for arable land, and jeopardizes food security (Jin et al., 2015).

Oils derived from microorganisms, alternatively known as single-cell oils (SCOs), are similar in composition to vegetable oils and animal fats (Carvalho et al., 2015). However, single-cell oils are preferred to plant- and animal-derived oils because it is easy to scale up their production. Also, the production of SCOs is not impacted by factors such as seasonal changes, geographic location, harvest time and transport, which are of concern when using plant and animal materials (Abghari and Chen, 2014; Dong et al., 2016). Single-cell oils are intracellular storage lipids made of triacylglycerols (TAGs), free fatty acids, polar lipids, sterols, hydrocarbons, and pigments (Carvalho et al., 2015; Ochsenreither et al., 2016). All organisms produce SCOs as part of their structural and functional processes, such as forming permeable membranes of cells and organelles (Ochsenreither et al., 2016). Nevertheless, the capacity of microorganisms to produce SCOs can vary from less than 1% to more than 80% (w/w) in dry cell weight (Athenaki et al., 2017). A small number of microorganisms are known as oleaginous microorganisms due to their unique ability to accumulate lipids over 20% (w/w) of their dry cell mass (Carvalho et al., 2015; Dong et al., 2016; Ochsenreither et al., 2016). The ability of oleaginous microorganisms to accumulate large amounts of lipids compared to non-oleaginous microorganisms is not due to a difference in fatty acid biosynthesis (Ochsenreither et al., 2016). Oleaginous microorganisms depend on a continuous supply of acetyl coenzyme A (acetyl-CoA) and nicotinamide adenine dinucleotide phosphate (NADPH) for the production of fatty acids through a reversed β-oxidation under nitrogen-limiting conditions (Ochsenreither et al., 2016). The propensity of microorganisms to accumulate lipids is also primarily determined by their genetic profile, and this may vary even among species or between strains of a given species (Athenaki et al., 2017).

As the body of knowledge is continually expanding in SCO research, it is imperative to update the scientific community with recent advancements. Irrespective of the expansion in SCO research efforts, filamentous fungi remain the less explored SCO producer. However, oleaginous filamentous fungi exhibit several desirable intrinsic features such as a repertoire of hydrolyzing enzymes and a unique pellet morphology that facilitates downstream harvesting, prompting the need for the present work. Hence, this review examines the ability of filamentous fungi in the hydrolysis of various organic feedstocks to produce SCOs. This review also explores the types of mechanisms regulating the synthesis of SCOs from these carbon materials. Furthermore, the paper evaluates the possibilities of integrating SCO processes with other biotechnological processes such as bio-hydrogen production. Future prospects of SCO fermentations are also discussed.

## Overview of Filamentous Fungal SCOs for Biodiesel Production

Identifying microorganisms capable of producing a significant amount of SCOs, with compositional similarities to conventional lipids, will be highly beneficial for improving process economics (Athenaki et al., 2017). *Mucor circinelloides* were the first filamentous fungi used in the commercial production of polyunsaturated fatty acids (PUFAs) (Ratledge, 2004; Čertík et al., 2012). Single-cell oils produced by oleaginous microorganisms such as filamentous fungi are desirable as starting material for biodiesel production due to their high degree of unsaturation. Unfortunately, the large-scale industrialization of microbial SCO has not been achieved due to the high cost of production. Over the years, innovations such as introducing novel fermentation technologies and versatile engineered oleaginous strains have improved the competitiveness of microbial lipid production. Despite all these developments, microbial lipids’ production cost remains high and needs to be reduced to realize its economic viability for use in biodiesels, drop-in biofuels, and other products (Abghari and Chen, 2014).

One strategy that may be exploited to lower PUFA production costs is to integrate microbial oil production with other high-value compounds in a process that uses low-cost agro-industrial waste and lignocellulosic biomass (Ratledge, 2004). Since filamentous fungi produce various hydrolyzing enzymes, allowing them to metabolize complex substrates (Gmoser et al., 2019), they can produce PUFAs from such low-cost substrates. Thus, the use of filamentous fungi as SCO producers presents an opportunity to combine saccharification and fermentation for efficient use of low-cost substrates, such as agro-industrial waste and lignocellulosic substrates, making the process more cost-effective (Martínez et al., 2015).

## Oleaginous Filamentous Fungi: Diversity, Screening and Lipid Biosynthetic Pathway

### Diversity of Oleaginous Filamentous Fungi

**FIGURE 1:**
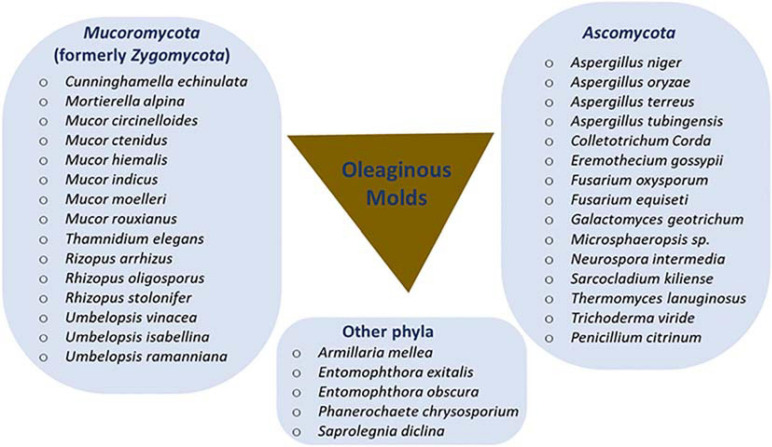
Phyla classification of oleaginous molds. Revised (previous names in parenthesis) taxonomic classifications include genera *Umbelopsis* (*Mortierella), Thermomyces* (*Humicola*), and *Eremothecium* (*Ashbya*) as well as the species *Mucor moelleri* (*Zygorhynchus moelleri*), *Mucor ctenidius* (*Thamnidium ctenidium*), and *Rhizopus arrhizus* (*Mucor rouxii*).

Several oleaginous filamentous fungi, mostly belonging to two major phyla (*Mucoromycota* and *Ascomycota*), have been identified and are taxonomically summarized in Figure 1. *Mucor circinelloides* was the first species used for the industrial production of γ-linolenic acid (GLA)-rich oil, a precursor for several biologically active compounds (Somashekar et al., 2003; Ratledge, 2013). Over the years, several other oleaginous filamentous fungi have been identified from different sources, including soil, herbaceous plants and animal feeds (Liu et al., 2010; Dey et al., 2011; Banu et al., 2017; Muniraj et al., 2017; Tonato et al., 2018). Particular interest has been placed on Mucoromycetes (formerly Zygomycetes) and Ascomycota species, such as *Aspergillus niger, Cunninghamella echinulata*, *Thermomyces lanuginosus* (formerly *Humicola lanuginosa*), *Umbelopsis isabellina* (formerly *Mortierella isabellina*), and *Umbelopsis vinacea* (formerly *Mortierella vinacea*), because of their high lipid accumulation (Papanikolaou et al., 2004; Fakas et al., 2008, 2009; Meng et al., 2009).

### Screening for Oleaginous Filamentous Fungi With Desirable SCOs

Single-cell oils derived from filamentous fungi are highly attractive feedstocks in sustainable biodiesel production since they produce SCOs with similar characteristics to those of conventional vegetable oils. Interestingly, biodiesel produced from microbial oils can be mixed with petroleum diesel at any ratio (Bhanja et al., 2014). Due to the array of benefits associated with filamentous fungi-derived SCOs for third-generation biodiesel production, there has been a surge in screening efforts for promising oleaginous strains (Khot et al., 2012; Abu-Elreesh and Abd-El-Haleem, 2014; Abou-Zeid et al., 2019). Primary considerations when selecting a fungal strain for biodiesel production include elevated total lipid yield, especially high levels of TAGs, and the optimal composition of fatty acids (Khot et al., 2012; Bardhan et al., 2019). In this regard, the degree of branching and unsaturation, as well as chain length, are notable structural features to be considered when selecting fungal SCOs for biodiesel production (Sitepu et al., 2014; Table 1). Oleaginous fungal strains that produce oils that are rich in monounsaturated fatty acids (MUFA) and saturated fatty acids (SFA) can be utilized for biodiesel production, whereas strains rich in polyunsaturated fatty acids (PUFAs) are more desirable for nutraceuticals and pharmaceuticals (Patel et al., 2020). This is due to lipid with elevated levels of PUFAs, which possess more than two double bonds, being susceptible to auto-oxidation. Hence, PUFA rich lipids are more unstable and technically unsatisfactory for biodiesel production. Moreover, PUFA rich lipids results in an unpleasant odor (Knothe, 2012; Patel et al., 2020).

**TABLE 1:** Relationship between structural features of fatty acids and selected biodiesel properties as well as biodiesel standards (Rashid et al., 2009; Sitepu et al., 2014; Singh et al., 2019).

	**Structural features of fatty acids**			**Desired property for biodiesel**	**Biodiesel standards**	
			
	**Degree of unsaturation**	**Chain length**	**Branching**		**EN 14214**	**ASTM D6751**
Cetane number	Saturated preferred	Longer chains preferred	NR	Influences ignition delay: Greater is better	>51 min	>47 min
Heating value/calorific value	NR	Longer chains preferred	NR	Indicates energy yield upon fuel combustion: Greater is better	>35 MJ/kg (in EN 14213)	NS
Oxidative stability	Saturated preferred	NR	NR	Measures degree of biodiesel reaction with air: More stable is better	At 110°C: > 6 h	At 110°C: > 3 h
Melting point	Unsaturated preferred	Shorter chains preferred	Branching preferred	Influences cold flow properties: Lower is better	NS	NS
Kinematic viscosity	Unsaturated preferred	Shorter chains preferred	NR	Influences penetration and atomization of fuel spray: Less viscous is better	at 40°C: 3.5–5.0 mm^2^/s	at 40°C: 1.9–6.0 mm^2^/s

*NR, not relevant; NS, not specified.*

In general, lipids intended as feedstock for biodiesel production must be composed of elevated concentrations of MUFAs such as palmitoleic acid (C16:1) and oleic acids (C18:1), controlled concentrations of SFAs such as palmitic acid (C16:0) and stearic acid (C18:0) and minimal quantities of PUFAs such as linoleic acid (C18:2) and γ-linolenic acid (C18:3) (Miglio et al., 2013). Fatty acid profiles influence various biodiesel properties such as melting point, cetane number, heating value/calorific value, kinematic viscosity and oxidative stability, which greatly affect the performance of the generated biodiesel (Sitepu et al., 2014). The above-mentioned biodiesel properties are regulated by standards (Table 1); hence the screening process must be conducted with utmost care to ensure that the selected strain is ideal for biodiesel production.

Fungal strains that produce lipids with the above-mentioned qualities and that can metabolize low-cost substrates, display a rapid growth rate, and can be easily harvested, are considered more valuable due to the ability of such strains to substantially reduce costs associated with the production process (Sitepu et al., 2014).

An array of detection methods has been established as the first step when screening oleaginous fungi for lipid production potential (Kosa et al., 2017). The second step in the screening process is frequently a qualitative evaluation of the lipids produced by the fungal strain of interest. Traditional methods such as gas chromatography-mass spectrometry (GC-MS) and gas chromatography-flame ionization detection (GC-FID) is mostly utilized for fatty acid analysis (Rumin et al., 2015).

Traditional oleaginous fungi screening methods, which are mostly based on cultivation of strains of interest in shake flasks followed by lipid extraction, quantification and qualitative analysis are feasible when screening a small set of fungal isolates. However, it becomes laborious, costly and inefficient when screening hundreds of strains (Forfang et al., 2017; Kosa et al., 2017). Therefore, high-throughput screening methods such as those described by Kosa et al. (2017) and Yao et al. (2019) have been developed to improve the efficiency of the screening process.

Since the selection of fungal strains with the highest lipid productivity is imperative to improve the cost-effectiveness of the entire biodiesel production process, the adoption of currently available high-throughput screening methods and the development of novel, more rapid screening methods will accelerate the oleaginous fungal screening process and enable the selection of the most promising strains for downstream application in biodiesel generation. Moreover, the integration of automation in the screening process will ensure that more fungal strains can be screened simultaneously, improving the probability of the most promising isolate being selected.

### Fatty Acid Biosynthesis in Oleaginous Filamentous Fungi

The synthesis of fatty acids in oleaginous microorganisms follows two metabolic routes, *de novo* (on hydrophilic substrates) or *ex novo* (on hydrophobic substrate) (Subhash and Mohan, 2011). In this review, we will focus only on *de novo* lipid synthesis route. The regulation of lipid biosynthesis has been extensively studied in other microorganisms such as yeast, but little has been reported on filamentous fungi. A better understanding of the cellular machinery regulating lipid metabolism in filamentous fungi is essential for improving lipid yields and fatty acid profiles (Laoteng et al., 2011; Vorapreeda et al., 2012; Chen et al., 2015). Although the lipid accumulation process seems to be conserved, there is a considerable variation amongst oleaginous species and even within strains of the same species (Rodríguez-Frómeta et al., 2013). The factors driving these variations are not yet known. Fortunately, genomic data is now available in the literature, and more studies have provided better insights into the regulation of lipid metabolism in oleaginous microorganisms (Vorapreeda et al., 2012).

Lipid biosynthesis involves multiple enzymes, including malic enzymes (MEs), malate transporter, desaturases, ATP-citrate lyase (ACL), adenosine monophosphate deaminase (AMPD), and acetyl-CoA carboxylase 1 (ACC1) (Hao et al., 2014, 2016; Shi et al., 2014; Kerkhoven et al., 2016; Zhao et al., 2016; Zhang et al., 2017; Chang et al., 2019; Wang et al., 2019). Importantly, NADPH and acetyl-CoA (fatty acid precursor) supply are crucial for lipid biosynthesis and accumulation in oleaginous microorganisms (Wang et al., 2019). The major pathways for the generation of NADPH during glucose metabolism include: (i) the pentose phosphate (PP) pathway, with glucose 6-phosphate dehydrogenase and 6-phosphogluconate dehydrogenase, (ii) the pyruvate/oxaloacetate/malate (POM) cycle, through NADP + dependent malic enzyme, or (iii) the tricarboxylic acid (TCA) cycle via NADP + dependent isocitrate dehydrogenase (Zhao et al., 2016; Wang et al., 2019).

**FIGURE 2:**
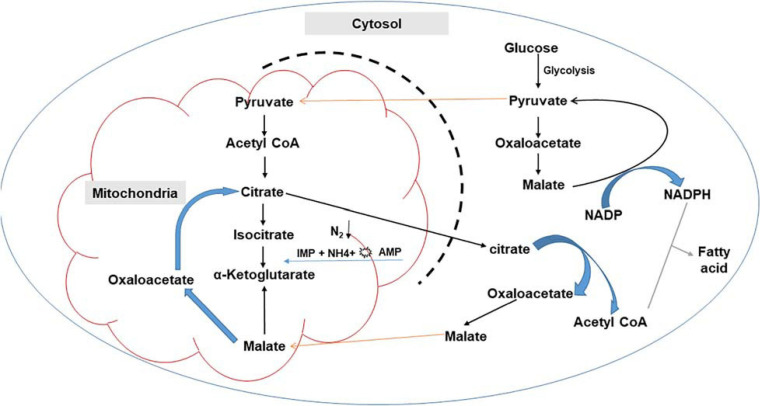
Schematic representation of fatty acid synthesis in oleaginous microorganisms.

Nutrient imbalances, most typically the excess of carbon source and a limitation in other vital nutrients such as nitrogen, triggers several physiological and metabolic changes leading to the channeling of the carbon flux toward lipid synthesis (Laoteng et al., 2011; Papanikolaou, 2011; Athenaki et al., 2017). Nitrogen starvation results in the activation of adenosine monophosphate (AMP) deaminase, which decreases the concentration of intracellular AMP (Garay et al., 2014; Athenaki et al., 2017). A decrease in mitochondrial AMP concentration alters the Krebs cycle function, resulting in a loss of activity by isocitrate dehydrogenase, which is allosterically activated by intracellular AMP (Papanikolaou, 2011; Tan et al., 2016; Dourou et al., 2018). Isocitrate accumulates in the mitochondria as the production of α-ketoglutarate in the tricarboxylic cycle (TCA) cycle is reduced (Garay et al., 2014). Isocitrate is converted back into citrate, resulting in the carbon flow being redirected toward intra-mitochondrial citric acid (Papanikolaou, 2011; Athenaki et al., 2017). Excess citrate is subsequently secreted to the cytoplasm by an antiport protein, citrate/malate transporters. These transporters facilitate the transport of malate from the cytosol into the mitochondrion in exchange for mitochondrial citrate moving into the cytosol, thus linking the TCA cycle in mitochondria and fatty acid biosynthesis in the cytosol (Ratledge and Wynn, 2002; Zhao et al., 2016; Yang J. et al., 2019). In the cytoplasm, citrate is cleaved by ATP-citrate lyase (ACL) into oxaloacetate (OAA) and acetyl-CoA, which together with NADPH generated in the cytosol, is required for lipid biosynthesis (Ratledge and Wynn, 2002; Zhao et al., 2016; Yang J. et al., 2019; Figure 2). In a study by Vorapreeda et al. (2012), it was observed that ACL was not present in non-oleaginous strains, suggesting that this enzyme only confers an additional route of acetyl-CoA synthesis in oleaginous strains. Similar results were reported by Chen et al. (2015), where they identified 23 lipogenesis-associated genes in the oleaginous fungus, *Mortierella alpina*, which shared a high similarity of expression patterns to other oleaginous microorganisms, but not with non-oleaginous *Aspergillus nidulans*.

Oxaloacetate is reduced by malate dehydrogenase to form malate in the cytosol, which is subsequently converted to pyruvate by malic enzyme (ME) (Rodríguez-Frómeta et al., 2013). Malic enzyme plays a critical role in lipid synthesis, and earlier studies suggested that this enzyme is a rate-limiting step in fatty acid synthesis (Hao et al., 2014). Comparative studies have since identified five putative genes encoding MEs. Three of these MEs are located in the mitochondria, while the other two are cytosolic (Rodríguez-Frómeta et al., 2013). An additional route for acetyl-CoA can also be through the generation of glycerol 3-phosphate in glycolysis, which is a building block for the synthesis of phospholipids and triglycerides (Vorapreeda et al., 2012) as well as through the amino acid pathway (Hao et al., 2014).

Nicotinamide adenine dinucleotide phosphate has the reducing power required to convert acetyl-CoA into fatty acids (Figure 2). In this conversion, acetyl-CoA is carboxylated by acetyl-CoA carboxylase (ACC) to form malonyl-CoA (Garay et al., 2014). ACC is a multifunctional complex with components including biotin carboxylase, carboxyltransferase and biotin carboxyl carrier protein domains. This enzyme plays a role in controlling the overall lipid biosynthesis (Laoteng et al., 2011). The condensation of acetyl-CoA and malonyl-CoA follows the carboxylation process by a multi-enzyme complex fatty acid synthase (FAS), which elongates the acyl-CoA chain (Ledesma-Amaro et al., 2014). Acyl-chains are then added to the growing fatty acid chain. This is followed by three successive reactions, namely, reduction, dehydration and reduction, after which saturated fatty acid chains, palmitic acid (16:0) or stearic acid (18:0) are generated as the final product (Muniraj et al., 2015; Santek et al., 2018).

Lipid biosynthesis is complex and cannot be sufficiently gaged through the analysis of single genes or pathways (Chen et al., 2015). Based on the relatively conserved fatty acid pathways in oleaginous microorganisms, the increasing availability of sequence data provides more insight into the molecular mechanisms involved in regulating fatty acid synthesis (Vorapreeda et al., 2012).

## Advances in Improving SCO Production in Oleaginous Microorganisms

Lipid accumulation (the metabolic balance between lipid biosynthesis and degradation) in oleaginous molds can be improved by optimizing culture conditions and substrates and through metabolic engineering. In this section, we provide a review of notable studies in which optimization of cultivation conditions and engineering of genetic components and regulatory mechanisms in the genome of oleaginous filamentous fungi have been conducted in order to increase lipid accumulation.

### Effect of Culture Conditions on SCO Accumulation and Saturation

Storage lipids of oleaginous fungi usually contain TAGs with the right proportion of saturated fatty acids (SFA) during the stationary phase (Aggelis et al., 1995). The lipids are stored within specialized organelles known as lipid granules/lipid droplets. The process of lipid formation in the cell involves the metabolic conversion of the external carbon source into carbohydrates or hydrocarbons and subsequently, lipids (Subhash and Mohan, 2011). Various types of polysaccharides derived from agro-industrial activities are used as carbon sources for SCO production. However, carbon sources such as glucose and starch, which have fundamental biochemical similarities, can lead to differences in lipid accumulation when used for the cultivation of certain oleaginous filamentous fungi (Dyal et al., 2005; Chatzifragkou et al., 2010). Also, the metabolism of various individual sugars can impact the fatty acid composition of storage lipids (Somashekar et al., 2003; Jang et al., 2005; Papanikolaou and Aggelis, 2010).

Other factors which affect the accumulation and fatty acid composition of cellular lipids include temperature, pH, cultivation period, fortification of culture media with specific nutrients such as nitrogen, the inclusion of growth enhancers, agitation, aeration and incorporation of substrates at different concentrations and time points (Hansson and Dostálek, 1988; Kendrick and Ratledge, 1992b; Carvalho et al., 1999; Somashekar et al., 2003; Papanikolaou et al., 2004; Jang et al., 2005; Fakas et al., 2009; Liu et al., 2010; Dey et al., 2011). Some studies investigating optimal values of these cultural parameters for maximum lipid accumulation and fatty acid composition are summarized in Table 2. In most cases, values above or below the optimum led to lower lipid yield or different fatty acid composition. Importantly, the accumulation of lipids is influenced not only by individual factors but a combination of factors and is species/strain-specific. Thus, the optimization of process parameters for maximum lipid yields is usually required and conducted on a strain-by-strain basis (Table 3). Some of these factors that affect lipid accumulation and fatty acid composition are discussed in the following subsections.

**TABLE 2:** Effect of culture conditions on lipid accumulation in oleaginous molds.

**Factor/Strain**	**Experimental conditions/range**	**Optimum conditions for max. total lipid accumulation**	**At optimum conditions**			**References**
			
			**Biomass dry wt. (g/L)**	**Total lipid yield (Specific PUFA)**	**Degree of unsaturation (Δ/mole)**	
**Agitation/Agitation speed**						
*Mucor ctenidius* (*Thamnidium ctenidium*) SCIM 3.009	140–220 rpm	220 rpm	11.9	22.6 g/L	−	Liu et al., 2010
*Mortierella* sp. 1b	Shaking (at 250 rpm) vs. Static	Shaking at 250 rpm improved total lipid and GLA with a max on Day 6	∼8.0	∼35.0 g/L (Rel. GLA −12.4%)	−	Somashekar et al., 2003
*M. rouxii* 386	Shaking (at 250 rpm) vs. Static	Shaking at 250 rpm improved total lipid and GLA yield with a max on Day 6	∼6.0	∼32.0 g/L (Rel. GLA −13.8%)	−	Somashekar et al., 2003
*Trichoderma viride* NRC 314	Shaking (at 150 rpm) vs. Static for 5 days	Static	16.0	4.5 g/L	−	Ali and El-Ghonemy, 2014
Aeration/Aeration rate						
*Entomophthora exitalis* NRRL 3742	Varying aeration rate ranging from 150 to 1000 ml/min at fixed dilution rate (0.04 h^–1^) and temperature (22°C)	No difference across 150 to 1000 ml/min aeration rates	4–4.5	∼7.5% w/w DW	−	Kendrick and Ratledge, 1992b
*Galactomyces geotrichum*	Aeration at 0.5, 1.5, and 3.0 vvm for 48, 96, 168, and 214 h	1.5 vvm for 48 h	−	n−6 PUFA (1.2–1.4 mg/g) and n−3 PUFA (0.4–0.5 mg/g)	−	Grygier et al., 2020
**pH**						
*Mucor ctenidius* (*Thamnidium ctenidium*) SCIM 3.009	pH 4.5–8.5	pH 6.5	∼11.0	∼10.0 g/L	−	Liu et al., 2010
*Mortierella alpina* NRRL-A-10995	pH range 3 to 8 at intervals of 1. Exponential/Stationary growth phase	pH 5.0 at exponential growth phase/pH 7 at stationary growth phase	14.4/22.0	17.8 g/L (2.27 g/L AA)/19.4 g/L (2.67 g/L AA)	−	Mironov et al., 2018
*Mucor circinelloides* ATCC 1216B	Initial pH range of 3.0 to 10.0	pH 3.0	1.25	30.87% w/w DB	−	Xia et al., 2011
*Galactomyces geotrichum*	pH 4.5 and 6.5 for 48, 96, 168, and 214 h	pH 6.5 for 48 h and pH 4.5 for 214 h	−	Total PUFA 25.8–26.4 mg/g	−	Grygier et al., 2020
*Trichoderma viride* NRC 314	pH 3.0–pH 8.0	pH 4.0	∼12.5	∼3.5 g/L	−	Ali and El-Ghonemy, 2014
*Mucor circinelloides* ATCC 1216B	pH adjustment (between 3.0 and 8.0) with NaOH or CaCO_3_ powder (0.4 g/100 mL) at 18 h	Adjusted pH of 6.0 after 18 h of growth	∼4.0	∼8.00% w/w DB	−	Xia et al., 2011
*Mucor circinelloides* ATCC 1216B	pH adjustment to 5.3 at 6, 12, 18, 24, and 36 h using CaCO_3_	≥18 h. Highest lipids at 36 h	3.0	∼8.50% w/w DB	−	Xia et al., 2011
**Carbon-Nitrogen Ratio**						
*Cunninghamella echinulata* ATHUM 4411	C/N of 55 and 100 [mol/mol]	C/N ratio of 100 (mol/mol)	9.2	3.2 g/L	−	Papanikolaou et al., 2007
*Umbelopsis ramanniana* (*Mortierella isabellina)* ATHUM 2935	C/N of 55 and 100 [mol/mol]	C/N ratio of 100 (mol/mol)	10.9	5.2 g/L	−	Papanikolaou et al., 2007
*Mortierella alpina* ATCC 32222	2.5–48.3	C/N ratio of 5.2	7.20	1.41 g/L	3.79	Jang et al., 2005
*Colletotrichum* sp. (isolate DM06) and *Alternaria* sp. (isolate DM09).	40:1 to 240:1	C/N ratio of 240:1 in both strains		7.3 g/L (46.8%, w/w DW) and 7.6 g/L (55.1%, w/w DW), respectively	−	Dey et al., 2011
**Nitrogen source and concentration**						
*Mortierella alpina* ATCC 32222	NH_4_NO_3_, NaNO_3_, KNO_3_, (NH_4_)_2_SO_4_, yeast, urea	Mixture of KNO_3_ and yeast extract at 2:1 (w/w)	7.20	1.44 g/L	3.87	Jang et al., 2005
*Mucor ctenidius* (*Thamnidium ctenidium*) SCIM 3.009	Inorganic (NH_4_NO_3_, NaNO_3_, KNO_3_ and (NH_4_)2SO_4_) and organic (peptone, yeast extract, corn powder, soybean and wheat bran) nitrogen source	NH_4_NO_3_	17.5	9.1 g/L	−	Liu et al., 2010
*Rhizopus arrhizus* (*M. rouxii*) 386	KNO_3_, NH_4_Cl, NH_4_NO_3_, (NH_4_)_2_SO_4_, NH_2_CONH_2_	KNO_3_ – highest PUFA yield	∼7.0	∼32.5 g/L (Rel. GLA −13.8%)	−	Somashekar et al., 2003
*Mortierella* sp. 1b		KNO_3_ – highest PUFA yield	∼7.0	∼30.0 g/L (Rel. GLA −12.4%)	−	Somashekar et al., 2003
*Mortierella ramanniana* CBS 112.08	KNO_3_, NH_4_Cl, (NH_4_)_2_SO_4_	KNO_3_ – highest PUFA	22.6	15.7% w/w DB	−	Hansson and Dostálek, 1988
*Colletotrichum* sp. (isolate DM06), *Alternaria* sp. (isolate DM09).	Peptone, (NH_4_)2SO_4_, NH_4_NO_3_ and KNO_3_	Peptone	−	−		Dey et al., 2011
*Mucor circinelloides* ATCC 1216B	0.77 to 11.21 mmol/L N	0.77 mmol/L N (highest C:N ratio of the experiment)	∼3.6	30% w/w DB	−	Xia et al., 2011
**Amount of Nitrogen + metals**						
*Mortierella ramanniana* CBS 112.08	KNO_3_ (0.5 – g/L) ± Cu, Zn, Mg, Mn	KNO_3_, 0.5 g/L, without metals	11.6	28.9% w/w DB	1.08	Hansson and Dostálek, 1988
**Temperature**						
*Mucor circinelloides* ATCC 1216B	24 to 30°C	24°C	∼1.6	∼21% w/w DB	−	Xia et al., 2011
*Mucor ctenidius* (*Thamnidium ctenidium*) SCIM 3.009	20 to 35°C	30°C	∼8.0	∼9.0 g/L	−	Liu et al., 2010
*Entomophthora exitalis* NRRL 3742	Between 18 and 30°C, pH 6.0	30°C	−	20% w/w DB (w/w, 8% AA, 90% TAG, and 10% DAC)	−	Kendrick and Ratledge, 1992b
Rhizopus arrhizus (*Mucor rouxii*) CFR-G15	Between 5 and 40°C at 5°C intervals	30°C	∼7.5	∼38% w/w DB	−	Mamatha and Venkateswaran, 2010
*Mucor circinelloides*	25–45°	37°C	20.0	−	−	Mitra et al., 2012
*Trichoderma viride* NRC 314						
*Mortierella alpina* ATCC 32222	12–25°C	Temperature at 12°C (initial pH 6.0; basal medium)	6.20	1.63 g/L	3.87	Jang et al., 2005
*Mucor* sp. LB-54	5 to 45°C	Temperature of 28°C; 5 days	5.83	20.73 g/L (GLA, 43.97 g/L)	−	Carvalho et al., 1999
*Colletotrichum* sp. (isolate DM06), *Alternaria* sp. (isolate DM09).	−	28°C	−	−	−	Dey et al., 2011
*Trichoderma viride* NRC 314	20–45°C	28°C	16	4.66 g/L	−	Ali and El-Ghonemy, 2014
**Temperature and cultivation time**						
*Mortierella ramanniana* CBS 112.08	20–30°C for 3 and 6 days	30°C for 6 days	5.9	25.1% w/w DB	1.08	Hansson and Dostálek, 1988
**Incubation period**						
*Trichoderma viride* NRC 314	24, 48, 72, 96, and 120 h. Constant pH 6.0 at 28°C	5 days (120 h)	∼19.0	∼6.0 g/L	−	Ali and El-Ghonemy, 2014
**Substrate/Substrate concentration**						
*Mucor ctenidius* (*Thamnidium ctenidium*) SCIM 3.009	20–100 g/L	60 g/L lactose	∼6.0	∼8.0 g/L	−	Liu et al., 2010
*Mortierella alpina* ATCC 32222	0–120 g/L	Soluble starch at 100 g/L (10% w/v)	18.4	3.97 g/L	3.88	Jang et al., 2005
*M. isabellina* ATHUM 2935	0–100 g/L	101.9 g/L	35.9	18.1 g/L	−	Papanikolaou et al., 2004
*Trichoderma viride* NRC 314	Dextrose conc. 10–110 g/L	50 g/L	∼20.0	∼7.0 g/L	−	Ali and El-Ghonemy, 2014
*Trichoderma viride* NRC 314	C_5_–C_18_ sugars	Dextrose	∼13.0	∼5.0 g/L	−	Ali and El-Ghonemy, 2014
*Trichoderma viride* NRC 314	PDLM, CDM, PDYE, etc.	PDLM	16.0	4.7 g/L	−	Ali and El-Ghonemy, 2014
**Fermenter type and cultivation time**						
*Mortierella ramanniana* CBS 112.08	Tower fermentor; fixed cultivation time	Tower fermentor; 92 h cultivation	6.4	24.4% w/w DB	−	Hansson and Dostálek, 1988
*Mortierella ramanniana* CBS 478.63	Tower fermentor; fixed cultivation time	Tower fermentor; 92 h cultivation	5.5	18.4% w/w DB	−	Hansson and Dostálek, 1988
*Mortierella vinacea* CBS 212.32	Tower, tank reactor (Turbulence by air mixing), stirred tank reactor (at 200 rpm); cultivation time of 45– 162 h	Tower fermentor; 92 h	29.5	15.6% w/w DB	−	Hansson and Dostálek, 1988
**Basal medium + glucose + Temperature**						
*Mucor* sp. LB-54	Basal medium w or w/o glucose (7 to 10% w/v) at 28 for 5 days and for incubation at 12°C for 3 days	Basal medium + 7% (w/v) glucose at 28°C (for 5 days) + 12°C (for 3 days)	6.27	25.07 g/L (GLA, 74.10 mg/L)	−	Carvalho et al., 1999
**Media and agitation**						
*Armillaria mellea* ATCC 11 114	Solid media (MEA and MWA) vs. liquid media (MEB and MW) w or w/o agitation at 115 rpm. W or w/o Growth stimulators and peptone	MEA w/o agitation	0.7 g/g carbohydrate	−	−	Hansson and Dostálek, 1988
**Inoculum preparation and density**						
*Cunninghamella echinulata* CCRC 31840	Vortexing time at maximum speed for 30, 60, 120, 180, and 240 s. Mycelia weight of 0.2, 0.4, and 1.0	240 s of vortexting and 1.0 g of mycelium	26.1	7.65 g/L	−	Chen and Liu, 1997
*Cunninghamella echinulata* CCRC 31840	Inoculum ratio of 1–5% + 0.2 g mycelia + 30 s vortex; and Inoculum ratio of 1–5% + 1.0 g mycelia + 4 min vortex time	4% inoculum, 1.0 g mycelia and 4 min vortex	38.1	11.5 g/L (GLA, 1.337 mg/L)	−	Chen and Liu, 1997

*DW, Dry weight; GLA, gamma(γ) -linolenic acid; MEA, malt extract agar; MEB, malt extract broth; MW, modified Weinhold broth; MWA, modified Weinhold agar; DB, dry biomass; AA, arachidonic acid; TAG, triacylglycerols; DAG, diacylglycerols. –, not reported; PDLM, potato-dextrose liquid medium; CDM, Czapek Dox’s medium; PDYE, Potato-dextrose yeast extract.*

**TABLE 3:** Optimal culture conditions employed for maximum lipid production in some oleaginous filamentous fungal studies.

**Strain(s)**	**Carbon substrate/Culture medium**	**Inoculum density**	**C/N**	**Initial pH**	**Temp. (°C)**	**Cultivation period (hour)**	**Agitation speed (rpm)**	**Aeration rate**	**Culture mode**	**Medium/working vol.**	**Lipid yield/content**	**References**
*Mortierella alpine* ATCC 32222	10% of **soluble starch**, mixture of KNO3 and yeast extract at 2:1 (w/w) at C/N ratio 5.2–9.0 and 1% of **linseed oil**,	5% mycelial suspension of 1.0–1.5 × 10^6^ cells per mL	5.2	6.0	20°C (72 h) + 12°C (120 h)	72 + 120	200	−	Batch	−	Total PUFA of 943.2 mg/g carbon	Jang et al. (2005)
*Entomophthora exitalis* NRRL 3742	Semi-defined medium containing 15g/L **glucose**	−	−	6.0	30°C	−	−	2 L air/min	Batch	4 L	25% (w/w) DB	Kendrick and Ratledge (1992b)
*Trichoderma viride* NRC 314	PD liquid medium supplemented with 5% (50 g/L) **dextrose**	1 mL of 10^7^–10^8^ spores/mL	−	5.0	28°C	120	0	−	Batch	50 mL	43% (w/w) DB	Ali and El-Ghonemy (2014)
*Mucor ctenidius* (*Thamnidium ctenidium*) SCIM 3.009	60 g/L **glucose**, 3.0 g/L NH_4_NO_3_	10% (v/v) seed culture	−	6.5	30°C	100	220	1.5 vvm	Batch	3.5 L	13.6 ± 0.37 g/L (66.02%, w/w, DB)	Liu et al. (2010)
*Rhizopus arrhizus* (*Mortierella rouxii*) MTCC 3866 and *Mortierella* sp. 1b	Semisynthetic medium including 5g/L yeast. 30 g/L **soluble starch**, 1 g/L KNO_3_ and 20 mL/L **sesame oil**	−	−	5.5	28 ± 2°C	144	250	−	Batch	250 mL	44.5 and 49.83% (w/w) DB, respectively	Somashekar et al. (2003)
*Umbelopsis isabellina* (*Mortierella isabellina*)	68.1 g/L commercial **glucose**.	100 mL of 24-h culture	200	6.0	28°C	160	250–300	0.5 vvm; DOC > 20% v/v	Batch	2 L	∼12.5 g/L	Chatzifragkou et al. (2010)
*Galactomyces geotrichum Strain* 38	10 g/L **rapeseed oil**, 5 g/L yeast extract, 0.3 g/L metal salts and 10 mg/L vitamin B12 in 2.7 L volume	300 mL	−	6.5	30°C	214	100	1.5 vvm	Batch	3 L	23.65 mg/g	Grygier et al. (2020)
*Mortierella alpina* NRRL-A-10995	60 g/L **glycerol**, 5.0 g/L yeast extract and 3.30 g/L metal salts added at 5 mL/h	−	−	6.0	20°C	336	180–200	pO_2_ of 10–50%	Batch/Continuous	2.0 L	AA 25% (w/w) of lipids	Mironov et al. (2018)
*Cunninghamella echinulata* ATHUM 4411 and *Mortierella isabellina* ATHUM 2935	80 g/L **Xylose**	−	285	6.0	28°C	192 and 216, respectively	180	−	Batch	50 mL	57.7 and 65.5% (w/w) DB, respectively	Fakas et al. (2009)
*M. circinelloides* ATCC 1216B	20 g/L **glucose,** 1 g/L yeast extract, 1.5 g/L NH_4_Cl, 6g/L KH_2_PO_4_ and 1.2 g/L MgSO_4_ 7H_2_O	1.16 × 10^4^ spores/L	865.8	3.0	20–25°C	144	180	−	Batch	100 mL	−	Xia et al. (2011)
*Mucor circinelloides f. lusitanicus CBS 277.49*	Sterilized **whole thin stillage** (Total solids = 6.1%) from Corn-ethanol plant	10% (v/v) [prepared from 0.2% (v/v) of a ∼8 × 10^4^ spores/mL. suspension in 500 mL YM]	−	6.0	37°C	48	−	1.4 vvm	Batch	5 L	46% (w/w) DB	Mitra et al. (2012)
*M. isabellina* ATHUM 2935	101.9 g/**L glucose,** 0.5 g/L each of (NH_4_)_2_SO_4_ and yeast extract	1 ml of spore suspension (∼1–3 × 10^5^ spores)	340	6.0	28°C	250 h	170	−	Batch	50 mL	18.1 g/L	Papanikolaou et al. (2004)
*Cunninghamella echinulata* ATHUM 4411, *Mortierella ramanniana* MUCL 9235 and *Thamnidium elegans* CCF-1465	25 g/L **glycerol**	2 × 10^4^ spores/mL	−	6.0	28°C	144 h	180	−	Batch	50 mL	37.9, 44.1, and 37.2% (w/w) DB, respectively	Bellou et al. (2012)
*Mucor sp.* LGAM 36 and *Zygorhynchus moelleri* MUCL 143	25 g/L **glycerol**	2 × 10^4^ spores/mL	−	6.0	28°C	48 h	180	−	Batch	50 mL	37.7 and 48.4% (w/w) DB, respectively	Bellou et al. (2012)
*Cunninghamella echinulata* ATHUM 4411 *and Mortierella isabellina* ATHUM 2935	30 g/L commercial **glucose,** 0.5 g yeast extract,	1 mL (1 × 10^4^–2 × 10^4^ fungal spore suspension)	100	6.0	28°C	410 and 95, respectively	180	−	Batch	50 mL	3.2 and 5.2 g/L, respectively	Papanikolaou et al. (2007)
*M. isabellina NRRL 1757*	**Glycerol**, **orange peel waste** or **Ricotta cheese whey**	−	25–307	5.5–5.8	30°C	72–96	450	1.5 vvm; DOC of 100%	Batch	2 L	1.6–3.72 g/L	Carota et al. (2018)
*Aspergillus niger* LFMB 1 and *Aspergillus niger* NRRL 364	60 g/L **crude glycerol,** 0.5 g/L both of yeast extract and (NH_4_)_2_SO_4_	1 mL of a 1–3 × 10^5^ spore/mL suspension	−	6.0	28°C	140 and 165, respectively	240	DOC > 20% (v/v)	Batch	600 mL	1.4 and 2.5 g/L, respectively	André et al. (2010)
*Colletotrichum* sp. DM06 and *Alternaria* sp. DM09	100 g/L **Glucose**; 1.8 g/L peptone	I mL of ∼5 × 10^5^ spores/mL	160	−	28°C	240	150	−	Batch	50 mL	7.8 and 8.6 g/L	Dey et al. (2011)
*Colletotrichum* sp. DM06 and *Alternaria* sp. DM09	**Rice straw** and **wheat bran** (1:1 w/w)	Precultured 0.5 g wheat grains with mycelia	−	−	28°C	192	0	−	Batch	10 g substrates in 250 mL flask	68.2 and 60.32 mg/gds, respectively	Dey et al. (2011)
*Aspergillus terreus*	1% (w/v) **sugarcane bagasse** residue obtained after acid-pre-treatment; 1.5 g/L	I mL of 1–3 × 10^8^ spores/mL	−	Neutral pH	30°C	72 h	120	−	Batch	100 mL	1.72 g/L	Kamat et al. (2013)
*Phanerochaete Chrysosporium* ATCC 24725	**Wheat bran**, **corn straw** and **glucose mixture** (1:1:2, w/w)	1.0 g/bottle dry weight	−		30°C	216	0	−	Batch	200 mL	>40% (w/w) DB	Liu et al. (2019)
*Mucor circinelloides f. lusitanicus* ATCC 1216B	Hydrolyzed whey permeates	5% (v/v) preculture (1% spores in 250 mL PDB)	−	4.5	33.6°C	120	450	1 vvm	Batch	4 L	3.1 g/L/32% (w/w) DB	Chan et al. (2020)
*Mucor circinelloides* URM 4182	**Sugarcane molasses** (40 g/L sugar) supplemented with minor (synthetic) nutrients	7 × 10^7^ spores per batch	−	4.6	26°C	120	250	1.5 vvm	Batch	0.7 L	3.25 g/L/26% (w/w) DB	Bento et al. (2020)
*Aspergillus tubingensis* TSIP9	Palm empty fruit bunches + palm kernel cake (1:1, w/w)	0.85 mL of spore suspension containing 10^7^ spores/gds	−	−	28°C	120	−	−	Fed-Batch, every 3 days at 50% FM replacement	1 g + 1 mL	91.9 mg/gds	Cheirsilp and Kitcha (2015)
*Mortierella isabellina* ATHUM 2935	Rice hull hydrolyzate	1 mL spore suspension	57	6–6.4	28°C	∼230	180	40% (v/v) DOC	Batch	50 mL	3.6 g/L (64.3%, w/w DB)	Economou et al. (2011)
*Mucor circinelloides* URM 4182	Cheese whey	−	−	4.5	26°C	120	250	1.5 vvm	Batch	0.7 L	1.06 g/L (22.5%, w/w DB)	Braz et al. (2020)
*Mucor circinelloides* URM 4182	Vinasse and Molasses (40 g/L sugar) ratios	10^6^ spores/mL	−	4.5	26°C	120	250	1.5 vvm	Batch	0.7 L	25.9 wt.% of lipids	Reis et al. (2020)
*Aspergillus* sp. + microalgae (*Cholera vulgaris*) at a microalgae to fungal ratio of 100	Pre-treated molasses wastewater	1.5 × 10^4^/mL fungal spores		−	35°C	120	80	−	Batch	100 mL	4.215 g/L	Yang L. et al. (2019)

*DOC, dissolved oxygen concentration; –, not reported or not applicable; AA, arachidonic acid; DB, dry biomass (lipid in dry biomass); pO_2_, partial pressure of oxygen; PDB, potato dextrose broth; gds, gram per dry substrate; FM, fermented medium; vvm, volume of air per volume of medium per minute.*

#### Temperature

Temperature is a vital factor in regulating fungal fatty acid composition (Neidleman, 1987; Kendrick and Ratledge, 1992b; Mamatha and Venkateswaran, 2010). Lipid fluidity, consequent on the degree of unsaturation, can be influenced by the incubation temperature. Generally, at lower growth temperature, the proportion of unsaturated acids in the total lipid tends to increase (Carvalho et al., 1999). Hence, thermophilic fungi can produce fatty acids that are more saturated than mesophilic fungi (Bruszewski et al., 1972; Kendrick and Ratledge, 1992b). At lower temperatures, thermophilic fungi cannot produce unsaturated fatty acids in sufficient quantities to keep cell membranes in a liquid crystalline state (Neidleman, 1987; Kendrick and Ratledge, 1992b). An early study by Deven and Manocha (1976) on *Choanephora cucurbitarum* showed that there was increased production of γ-linolenic acid (GLA) at lower growth temperatures with a corresponding increase in the degree of unsaturation of total lipids observed. Kendrick and Ratledge (1992b) investigated the effect of varying temperatures (20–30°C) on fatty acid composition in the oleaginous filamentous fungus, *Entomophthora exitalis*, under continuous culture conditions with a constant dilution rate of 0.04 h^–1^. It was observed that the proportion of PUFAs in the total lipids increased proportionally from 18 to 27% (w/w) as the temperature decreased from 30 to 20°C. The observed increase in unsaturation was due to an increase in *n*−6 PUFA in the phospholipid and sphingo- plus glycolipid fractions. However, the growth of the fungus at a constant dilution rate and temperature (22°C) over a range of dissolved oxygen tension values did not influence lipid unsaturation and triacylglycerol fraction of the lipids. Thus, it was concluded that the observed change in lipid unsaturation is mainly a result of temperature.

Similarly, a study by Mamatha and Venkateswaran (2010) also concluded that temperature is the principal regulatory factor in the degree of unsaturation in the lipid profile of *Mucor rouxii* CFR-G15. In a separate study, Carvalho et al. (1999) observed that the strain *Mucor* sp. LB-54 accumulated intracellular lipid at 20.73% of dry cell weight, with a GLA content of 15% of total fatty acids, after 5 days of cultivation at 28°C. However, the GLA percentage increased to 24% of total fatty acids as the incubation temperature was reduced to 12°C. In general, as part of their adaptive response to low temperatures, oleaginous filamentous possess relatively high degrees of unsaturation in their lipid profile at lower temperatures (Mamatha and Venkateswaran, 2010).

#### Nitrogen

Nitrogen plays a crucial role in stimulating lipid accumulation. The effect of nitrogen is inverse as the accumulation of lipids by oleaginous fungi is triggered by the exhaustion of the exogenous nitrogen in the culture medium (Chatzifragkou et al., 2010; Xia et al., 2011). However, although the exhaustion of nitrogen allows the conversion of carbon to lipid, it prevents cell proliferation (Somashekar et al., 2003; Dey et al., 2011; Xia et al., 2011). Xia et al. (2011) observed that an increase in the initial nitrogen level in the culture medium coincided with a gradual increase in biomass and glucose consumption by *M. circinelloides*. However, in media with the highest carbon-to-nitrogen ratio, depletion of nitrogen by *M. circinelloides* caused the highest lipid accumulation of close to 30% of the dry weight. Dey et al. (2011) evaluated the biomass and lipid yields of two endophytic fungi, *Colletotrichum* sp. and *Alternaria* sp., using organic (peptone) and inorganic [(NH_4_)_2_SO_4_, NH_4_NO_3_, and KNO_3_] nitrogen sources. It was observed that nitrogen source influenced biomass and lipid yields of both, with the organic peptone source producing the highest biomass and lipid yield.

In contrast to Liu et al. (2010) and Dey et al. (2011) observed that NH_4_NO_3_ was the most suitable nitrogen source for highest biomass and lipid yields in *Mucor ctenidius* (*Thamnidium ctenidium*), compared to other inorganic and organic nitrogen sources such as yeast extract, wheat bran, peptone, soybean and corn powder. Elsewhere, KNO_3_ was the nitrogen source that gave the highest PUFA yield in *Mucor rouxii*, *Mortierella* sp., and *Mortierella ramanniana* strains (Hansson and Dostálek, 1988; Somashekar et al., 2003). The disparity in the suitability of different nitrogen sources observed in the above-highlighted studies can be attributed to strain-specific differences in nutrient requirements as well as differences in other growth conditions employed between studies.

#### pH

Studies have shown that pH is a stress-inducing factor that affects the accumulation of lipids and their PUFA profiles (Xia et al., 2011; Mironov et al., 2018). In a study by Xia et al. (2011), differences in pH [either acidic (pH 1–3) or alkaline (pH 8.0–10.0)] significantly influenced the cell growth and thus, lipid yields of *M. circinelloides*, with the highest lipid accumulation observed at a pH of 6.0. Cell growth was totally inhibited at pH values of 1 and 2. Mironov et al. (2018) investigated the effect of pH on the growth of *Mortierella alpina* (NRRL-A-10995) and the synthesis of lipids and arachidonic acid (AA) in both exponential and stationary phases of growth. The *M. alpina* strain was initially grown at pH 6.0 for 4 days, after which the pH was adjusted to a pH of 3, 4, 5, 6, 7, or 8 for a further 7 and 14 days. The authors observed that an optimal pH for the growth (biomass accumulation) of *M. alpina* was 5. Although biomass accumulation remained high between a pH of 5 to 7, a strong inhibition of growth was observed at a pH of 8 as well as at a pH below 5, with a pH of 3 strongly inhibiting the growth of *M. alpina*. Furthermore, the authors observed that AA synthesis was sensitive to an acidic pH and was inhibited at a pH of 3 (coinciding with inhibition of growth). These observations confirm that pH is a stress factor for cell growth and consequently, lipid accumulation and fatty acid composition.

#### Agitation and Aeration

Filamentous fungi are obligate aerobes; thus, oxygen is required for their cellular functions. Replenishing oxygen in the culture medium is achieved through aeration and/or agitation. Agitation helps to increase surface area for oxygen transport and concentration (dissolved oxygen). However, the rate of agitation needs to be optimized because vigorous agitation may lead to the shearing of cells, while a very low agitation rate may not improve the oxygen concentration sufficiently. Ali and El-Ghonemy (2014) investigated the effect of agitation (at 150 rpm) or the lack thereof on biomass and lipid accumulation of *Trichoderma viride*. They observed that the highest lipid accumulation (18.35%, w/w dry mass) occurred under static conditions compared to shake cultures (13.89%, w/w dry mass). In contrast, Somashekar et al. (2003) reported that an agitation speed of 250 rpm improved total lipid and GLA yields in both *Mortierella* sp. and *Mucor rouxii*. Similarly, lipid accumulation in *Mucor ctenidius* was higher at an agitation speed of 220 rpm compared to speeds below 220 rpm (Liu et al., 2010). Elsewhere, Kendrick and Ratledge (1992b) observed no difference in lipid accumulation across aeration rates of 150 to 1000 ml/min in *Entomophthora exitalis*, suggesting that aeration and aeration rates may not be as critical a factor as temperature for the accumulation of lipids in this fungus. Overall, the number of studies in which agitation improves lipid and PUFA profiles of oleaginous fungi appear to be higher in number than those reporting otherwise.

#### Substrate (Feedstock) Type and Concentration

Under nitrogen-limited conditions, excess carbon is diverted to lipid biosynthesis in many oleaginous filamentous fungi (Kavadia et al., 2001; Jang et al., 2005; Chatzifragkou et al., 2010). Carbon substrates are critical for both microbial nutrition, lipid accumulation and fatty acid composition of lipids (Somashekar et al., 2003). To this end, the effect of different carbon-rich substrates and their concentration on lipid accumulation in oleaginous molds have been investigated (Somashekar et al., 2003; Jang et al., 2005; Chatzifragkou et al., 2010; Ali and El-Ghonemy, 2014). Carbon sources (or substrates) used for lipid accumulation studies include molasses, sugar beet, soluble starch, and hydrolyzed carbohydrates such as glucose, sucrose, lactose, and raffinose.

Somashekar et al. (2003) investigated the relative percentage of fatty acids of lipids when *M. rouxii* and *Mortierella* sp. were cultivated on glucose, starch, sucrose and lactose. Among these carbon sources, GLA production was maximal in cultures grown on glucose, while lactose poorly promoted biomass and lipid production in both *M. rouxii* and *Mortierella* sp. Ali and El-Ghonemy (2014) reported that dextrose, compared to sucrose, lactose, fructose and other simple sugars, promoted the lipid accumulation in *Trichoderma viride*. The concentration of carbon source can also influence lipid accumulation in oleaginous filamentous fungi. Liu et al. (2010) investigated the effect of varying concentrations of lactose (20–100 g/L) on lipid accumulation in *Mucor ctenidius*. They observed that lipid and biomass yields increased steadily with lactose concentration, but peaked at 60 g/L, with higher concentrations causing a reduction in biomass and lipid yields.

In addition to carbon substrates, the supplementation with plant oils such as sesame oil, rapeseed oil and linseed oil, have been shown to increase biomass and lipid accumulation as well as alter polyunsaturated fatty acid profiles of lipids produced by oleaginous filamentous fungi (Somashekar et al., 2003; Jang et al., 2005; Grygier et al., 2020). In a study by Somashekar et al. (2003), the biomass and lipid accumulation of both *Mortierella rouxii* and *Mortierella* sp. grown on glucose supplemented with sesame oil were all higher than those grown on solely glucose. Surprisingly, there was a cessation of GLA production in the glucose plus sesame oil treatment, suggesting possible repression of Δ6-desaturase, an enzyme responsible for converting linoleic acid (LA, C18:2 ω6) to GLA. Similarly, Grygier et al. (2020) obtained maximum PUFA and higher levels of *n*−3 fatty acids when the medium was supplemented with 10 g/L rapeseed oil at optimum culture conditions than controls without plant oils. Jang et al. (2005) investigated the influence of soluble starch and glucose on lipid accumulation and fatty acid composition in *Mortierella alpina*. It was observed that arachidonic acid and total PUFAs production was maximal using 10% soluble starch and that further supplementation with linseed oil (1%, w/v) increased the total PUFA production.

Importantly, oleaginous microorganisms have several technical advantages compared to vegetable oil crops. However, the use of highly refined polysaccharides (i.e., industrial-grade glucose and sucrose) as carbon source may decrease the cost-effectiveness of SCO production in an industrial setting. It is estimated that substrates account for about 60 to 70% of the overall costs in fermentation processes (Papanikolaou and Aggelis, 2011; Tzimorotas et al., 2018). Therefore, carbon substrates derived from waste are now being explored to make this process both cost-effective and environmentally-friendly. To this end, studies utilizing waste materials as carbon sources have been investigated for microbial lipid production. These wastes include potato wastewater, orange peel extracts, sugarcane bagasse residues and cheese whey (Kamat et al., 2013; Muniraj et al., 2013; Carota et al., 2018; Braz et al., 2020).

For an efficient lipid production process, the carbon sources should consist of a high carbon-nitrogen (C/N) ratio (Ratledge, 1993). Several substrates were shown to have an appropriate C/N ratio for the lipid biosynthesis by fungal species. Carota et al. (2018) produced high lipid content in various oleaginous fungi (*Aspergillus*, *Mucor*, *Mortierella*, and *Cunninghamella*), using orange peel extract and ricotta cheese whey in shake-flasks. Amongst the tested strains, *Mortierella* exhibited superior performance and generated lipid productivity that ranged from 0.46–1.24 g/L.d. Muniraj et al. (2013) used potato wastewater for microbial lipid production by the oleaginous filamentous fungus, *Aspergillus oryzae*. In this study, maximum lipid production of up to 3.5 g/L.d was obtained at an optimum dilution ratio of 25%. The authors also observed that the major fatty acids in the lipids consisted of linolenic acid (5.5%), linoleic acid (6.5%), palmitic acid (11.6%), palmitolic acid (15.6%), and stearic acid (19.3%). More recently, sugarcane molasses was successfully used as sole carbon source to produce SCOs, using the filamentous fungus *Mucor circinelloides*. A maximum lipid productivity of 0.66g/L.d, which was equivalent to a lipid content of 29% (w/w) was attained (Bento et al., 2020). In a similar study, André et al. (2010) demonstrated an innovative approach of converting the biodiesel derived waste glycerol into value-added compounds (biomass, SCOs, and oxalic acid) using two fungal strains (*Lentinula edodes* and *Aspergillus niger*). In these experiments the authors used *Lentinula edodes* strains to produce biomass and two A. niger strains to produce SCO (3.1–3.5 g/L) and oxalic acid (20.5–21.5 g/L) under nitrogen-limiting shake flask conditions. In addition to these studies, other types of feedstocks that have been used in SCO production are shown in Table 2.

#### Inoculum

Inoculum density and size are factors that may influence lipid accumulation and fatty acid profile (Chen and Liu, 1997). High inoculum density may affect mycelial morphology, growth and product formation, by limiting the diffusion of nutrients within the mycelial floc (Chen and Liu, 1997). On the contrary, the growth of mycelium in smaller and compact inoculum pellets promotes biomass growth and lipid production, because the diffusion limitation of oxygen and other nutrients through the medium is reduced. The effects of inoculum on product formation have been reported in *Aspergillus oryzae* (Fukushima et al., 1991). Similarly, in a comprehensive study by Chen and Liu (1997), it was observed that the duration of vortexing (for cell homogenization) during inoculum preparation, the mass of the mycelial inoculum and suspension ratio influenced yields of biomass, lipids and GLA content of the lipids in *Cunninghamella echinulata*. Specifically, the maximum GLA yield of 1.349 mg/L was obtained at a vortex duration of 4 min, a mycelial mass of 1 g and an inoculum suspension of 4%.

### Genetic Engineering for Increased Lipid Accumulation in Oleaginous Filamentous Fungi

Over the years, genetic manipulation of oleaginous filamentous fungi for increased lipid accumulation has involved recombinant DNA technology through RNA interference (RNAi) (Takeno et al., 2005), electroporation (Gutiérrez et al., 2011) and vector-mediated transformations (Zhang et al., 2007; Ando et al., 2009; Chang et al., 2019; Khan et al., 2019a). Unlike in oleaginous yeasts, where the recently developed clustered regularly interspaced short palindromic repeats (CRISPR)/associated protein 9 (CRISPR/Cas9) system for gene editing has been applied to improve fatty acid accumulation (Yao et al., 2020), there is a paucity of studies utilizing CRISPR/Cas9 to engineer oleaginous filamentous fungi for increased lipid accumulation.

With the existing technologies, genetic modifications geared toward increasing lipid accumulation in oil-producing organisms have been performed using different strategies such as the overexpression of fatty acid biosynthetic enzymes, overexpression of TAGs biosynthesis-enhancing enzymes, regulation of related TAG biosynthesis bypass, partial blockage of TAG competing pathways and introduction of multiple genes which enhance lipid accumulation (Zou et al., 1997; Ratledge, 2002; Zhang et al., 2007; Lardizabal et al., 2008; Courchesne et al., 2009; Kamisaka, 2010; Liang and Jiang, 2013; Ledesma-Amaro et al., 2015; Table 4).

**TABLE 4:** Metabolic engineering strategies for increased lipid yields in oleaginous molds.

**Organism**	**Target gene/Gene product**	**Function of target gene or gene product**	**Strategy/Strategies**	**Comment on lipid/Fatty acid yield**	**References**
*Aspergillus nidulans*	Malic enzyme (malate dehydrogenase)	Metabolism of pyruvate and source of NADPH for *de novo* lipid biosynthesis and desaturation	Mutants lacking malic enzyme activity and elevated malic enzyme activity	Yield not significantly different from control under nitrogen limiting conditions	Wynn and Ratledge (1997)
*Ashbya gossypii*	*POX1* and *FOX1* gene	*POX1* and *FOX1* control the first and second steps of β-oxidation of fatty acids	Knock-out of *POX1* and *FOX1* genes (Blocking the β-oxidation Pathway)	Up to 70%, w/w, CDW in the recombinant compared to 15% CDW in the WT	Ledesma-Amaro et al. (2015)
*Ashbya gossypii*	CpFAH12/Δ12 desaturase	Conversion of oleic acid into linoleic acid	Heterologous expression of *CpFAH12*	20% w/w, CDW LA in the recombinant, amounting to 15-fold increase compared to the WT	Lozano-Martínez et al. (2016)
*Mortierella alpina*	Adenosine monophosphate deaminase (AMPD)	Breakdown AMP to IMP and NH_4_^+^ during nitrogen limitation	Overexpression of homologous AMPD	TFA in recombinant was 15.0–34.3% higher than control under N-limiting conditions	Chang et al. (2019)
*Mucor circinelloides*	Malate transporter	Transportation of cytosolic malate into the mitochondria in exchange for citrate moving into the cytosol	Overexpression of malate transporter gene	Increased the lipid content of about 70% [13 to 22% (w/w), CDW] compared to control strain.	Zhao et al. (2016)
*Mucor circinelloides*	Citrate transporter	Transportation of citrate from the mitochondria into the cytosol	Overexpression of citrate transporter genes	Increased lipid accumulation by 44–68% (18.8–21.8%, w/w, CDW)	Yang J. et al. (2019)
*Ashbya gossypii*	*OLE1* and *OLE2* which codes for Δ9 Desaturases	Insertion of the first double bond in palmitic and stearic acids by Δ9 desaturases	Simultaneous overexpression of *OLE1* and *OLE2*	Slightly increased TFA accumulation in glucose-based medium (up to 1.2-fold increase compared to WT)	Lozano-Martínez et al. (2016)
*Ashbya gossypii*	*AgMGA2* [coding for endoplasmic reticulum membrane protein (Mga2p)]	Regulation of *OLE1* which codes for Δ9 desaturases that insert the first double bond in stearic and palmitic acid to produce oleic and palmitoleic acid, respectively	Overexpression of *AgMGA2* which regulates *OLE1* encoding Δ9 desaturases	8.62% w/w, CDW on 8% (w/v) Glucose medium. This was a significant increase compared to WT.	Lozano-Martínez et al. (2017)
*Ashbya gossypii*	*AgFAA1* (*coding for* Acyl-CoA Synthetases)	Regulation of the intracellular concentration of the fatty acyl-CoA pool	Alteration of the fatty acyl-CoA pool by disruption of *AgFAA1*	Significant reduction in lipid accumulation, suggesting that FAA1 has other functions in addition to regulating intracellular levels of the fatty acyl-CoA pool.	Lozano-Martínez et al. (2017)
*Ashbya gossypii*	*AgTES1* and *ACOT5* (coding for acyl-CoA thioesterases)	Fatty acid degradation in the peroxisome	Alteration of fatty acyl-CoA pool by overexpression of *TES1* and *AC0T5*	Significant increase in TFA (up to two-fold) compared to WT. 118–122 mg/L FA in overexpression strains compared to 22 mg/L in WT	Lozano-Martínez et al. (2017)
*Ashbya gossypii*	*AgMGA2* [coding for endoplasmic reticulum membrane protein (Mga2p)] and *ACOT5* (coding for acyl-CoA-thioesterase)	Regulates *OLE1*; Fatty acid degradation in the peroxisome	Combination of a C-terminal truncated *AgMGA2* and overexpression of *AC0T5*	>20% w/w, CDW on 8% (w/v) Glucose after 7 days cultivation at 28°C	Lozano-Martínez et al. (2017)
*Mucor circinelloides*	Thioesterase	Metabolic control for the fatty acid chain length	Overexpression of thioesterase encoding genes	Reduction in chain length led to a 1.5 to 1.75-fold increase in total lipid productivity in recombinant strains	Hussain et al. (2019)
*Mortierella alpina*	ω3-desaturase	Catalyzing the conversion of n−6 fatty acid to n−3 fatty acid,	Overexpression of ω3-desaturase	Eicosapentaenoic acid (20:5n−3), reached a maximum of 40% of TFA	Ando et al. (2009)
*Mucor circinelloides*	*D15D* (Delta-15 desaturase)	Catalyses the production of stearidonic acid (18:4, n−3) from γ-linolenic acid (18:3, n−6)	Overexpression of delta-15 desaturase	Up to 5.0% stearidonic acid was accumulated	Khan et al. (2019a)
*Mucor circinelloides*	*fad3* gene (coding for fatty acid desaturase 3)	Catalyze the reactions for the production of stearidonic acid from linoleic acid (18:2, n−6)	Overexpression of *fad3* gene	Production of 340 mg/L stearidonic acid	Yang et al. (2020)
*Mortierella alpina*	Δ12-Desaturase	Desaturates oleic acid (18:1n−9) to linoleic acid (18:2n−6),	Silencing of Δ12-desaturase gene through RNA interference	Recombinant strains accumulated 18:2n−9, 20:2n−9, and mead acid (20:3n−9), which were not detected in both control and WT strains.	Takeno et al. (2005)
*Mucor circinelloides*	TE gene (encoding for thioesterase); the acyl-CoA oxidase (ACOX) and/or acyl-CoA thioesterase (ACOT) genes	Thioesterase, key enzyme governing the fate of fatty acid chain length; *ACOX* and *ACOT* play roles in fatty acid oxidation in the peroxisomes resulting in the degradation of long and medium-chain fatty acids for eventual utilization of acetyl-CoA as the sole carbon and energy source for growth	Combination of integration of heterologous acyl-ACP thioesterase (TE) into fatty acid synthase complex and subsequent disruption of acyl-CoA oxidase (ACOX) and/or acyl-CoA thioesterase (ACOT) genes with a preference for medium-chain acyl-CoAs	Mutant strains showed a significant increase in lipid production in comparison to the WT type strain. Medium-chain fatty acids were generally higher (up to 60%) in mutants compared to WT. Lipid productivity reached approx. 1.8 g/L per day.	Hussain et al. (2020)
*Aspergillus oryzae*	ATP-citrate lyase; fatty acid synthase; acetyl-CoA carboxylase; palmitoyl-ACP thioesterase	Fatty acid biosynthesis	Enhanced expression of genes encoding ATP-citrate lyase, fatty acid synthase, acetyl-CoA carboxylase and palmitoyl-ACP thioesterase using constitutively highly expressed *tef1* gene promoter	Enhanced expression of fatty acid synthase increased production of fatty acids and triglycerides by more than two folds. Overexpression of ATP-citrate lyase and palmitoyl-ACP only increased lipid productivity slightly while acetyl-CoA carboxylase overexpression did not increase productivity.	Tamano et al. (2013)
*Ashbya gossypii*	Endogenous xylose-utilization pathway genes *GRE3*, *XYL2*, and *XKS1*; Phopshoketolase pathway genes *PTA* (encoding for phosphotransacetylase) and *XPKA* (encoding for X5P phosphoketolase); and POX1 gene.	*GRE3, XYL2*, and *XKS1* confer xylose utilization to *A. gossypii*. PTA and XPKA are involved in the channeling of the carbon flux from xylose, through X5P, toward the pentose–phosphate pathway. *POX1* gene controls the control the first steps of β-oxidation of fatty acids	Overexpression of an endogenous xylose-utilization pathway genes (*GRE3, XYL2*, and *XKS1*); metabolic flux channeling from xylulose-5-phosphate to acetyl-CoA using a heterologous phosphoketolase pathway; and blocking of the *POX1* gene (involved in beta-oxidation of fatty acids)	Up to 69% lipid accumulation in recombinant strains harboring the three modifications compared to the parental control	Díaz-Fernández et al. (2017)
*Mucor circinelloides*	*G6pd* (encoding for glucose 6-phosphate dehydrogenase) and *6pdg* (encoding for 6-phosphogluconate dehydrogenase)	Pentose phosphate pathway for the provision of NADPH for lipid biosynthesis	Overexpression of *g6pd* and *6pgd*	Increased lipid content by 20–30% cell dry weight compared to the control strain	Zhao et al. (2015)
*Mucor circinelloides*	Malic enzyme	Metabolism of pyruvate and source of NADPH for fatty acid synthesis and desaturation	Overexpression of malic enzymes encoding genes from *Mucor circinelloides* and *Mortierella alpina in M. circinelloides*	Lipid content increased from 12% of the biomass to 30%. Concomitantly, degree of fatty acid desaturation increased slightly in recombinants	Zhang et al. (2007)
*Ashbya gossypii*	*ACC1* (codes for Acetyl-CoA carboxylase); *MGA2* (coding for endoplasmic reticulum membrane protein); DGA1	*ACC1* is the initial and rate-limiting enzyme of FA biosynthesis; *MGA2* is a regulator of the main Δ9 desaturase gene (*OLE1*). DGA1 codes for diacylglycerol acyltransferase	Overexpression of a feedback resistant form of the acetyl-CoA carboxylase enzyme; the expression of a truncated form of MGA2; and overexpression of an additional copy of DGA1 in *A. gossypii*	Mutant strain (harboring all three modifications) accumulated about 40%, (w/w), CDW in lipid content when grown on organic industrial waste	Díaz-Fernández et al. (2019)
*Mortierella alpina*	Glucose-6-phosphate dehydrogenase (G6PD); 6-phosphogluconate dehydrogenase (PGD): isocitrate dehydrogenase (IDH); malic enzyme (ME)	Generation of NADH for fatty acid biosynthesis and desaturation	Overexpression of individual genes and co-expression of *G6PD2* and *ME2* genes	Overexpression of *G6PD2* showed a 1.7-fold increase in TFA while *ME2* was more related to desaturation with a 1.5-fold increase in AA. Co-expression of *G6PD2* and *ME2* led to a 7.2-fold increase in AA within 96 h glucose fed-batch fermentation	Hao et al. (2016)
*Mortierella alpina*	Mitochondrial malic enzyme	Metabolism of pyruvate and source of NADPH for fatty acid synthesis and desaturation	Overexpression of mitochondrial malic enzyme	Arachidonic acid (20:4 n−6) content increased by 60% without affecting TFA	Hao et al. (2014)

*CDW, cell dry weight; WT, wild-type; LA, linoleic acid; FA, fatty acid; TFA, total fatty acids; AMP, adenosine monophosphate; IMP, inosine monophosphate; AMPD, adenosine monophosphate deaminase; AA, arachidonic acid.*

Lipid biosynthesis in oleaginous organisms is mediated by various gene products, which collectively work toward the synthesis of fatty acid by converting intracellular carbon. These genes and their associated proteins have been the target of many metabolic engineering studies. The NADP^+^-dependent malic enzyme (ME) has been shown to be a major source of NADPH which is critical for the synthesis of storage lipids in *Mucor circinelloides* (Zhang et al., 2007) and other oleaginous fungi (Evans and Ratledge, 1985; Kendrick and Ratledge, 1992a; Wynn and Ratledge, 1997). Zhang et al. (2007) utilized two recombinant strains of *M. circinelloides* in which the full length ME-coding genes from either *M. circinelloides* or *Mortierella alpina* were integrated under the control of the constitutive glyceraldehyde-3-phosphate dehydrogenase gene (*GPD1*) promoter. Upon cultivation of the two recombinant strains on a high C:N ratio medium in automated controlled submerged-culture bioreactors, a 2- to 3-fold increase in the activities of malic enzyme was observed, with a corresponding increase in the cellular lipid content from 12 to 30%. This increased lipid content coincided with a slight increase in the degree of fatty acid desaturation. These results confirm that the supply of NADPH solely by ME is a rate-limiting step during fatty acid biosynthesis in *Mucor circinelloides* and *Mortierella alpina*.

To elucidate the role of the citrate transporter in relation to lipid biosynthesis and accumulation, Yang J. et al. (2019) inserted citrate transporter genes, namely citrate transporter (*CT*) or tricarboxylate transporter (*TCT*), into *M. circinelloides* (CBS 277.49). Compared to the control, overexpression of *CT* or *TCT* caused an increase in lipid accumulation of 44% (from 13.0 to 18.8%, w/w, cell dry weight) and 68% (from 13.0 to 21.8%, w/w, cell dry weight), respectively. Moreover, Yang J. et al. (2019) observed that overexpression of the citrate transporter genes activated the downstream enzyme activities in lipid biosynthesis, indicating a greater flux of carbon toward fatty acid biosynthesis. In a separate study to elucidate the role of the malate transporter (MT) in relation to fatty acid synthesis and accumulation in *M. circinelloides*, Zhao et al. (2016) utilized both MT knockout and MT-overexpressing strains and found that, compared to the control, the lipid content in the overexpression mutant increased by approximately 70% (13 to 22% dry cell weight), while the lipid content in the knockout mutant decreased by 27% (13 to 9.5% dry cell weight). In a follow-up experiment, aimed at elucidating the mechanism by which the MT promotes the transportation of mitochondrial malate and citrate in relation to lipid accumulation, Wang et al. (2019) utilized ^13^C-labeled glucose metabolic flux analysis of the TCA cycle, glyoxylic acid (GOX) cycle and the pentose phosphate (PP) pathway in *M. circinelloides*. The authors found that the TCA cycle flux ratio of MT-overexpression strains decreased compared to that of the control strain, although the GOX cycle flux ratio was increased. As expected, a reverse trend was observed for the MT-knockout strain. Taken together, these observations suggest that the GOX cycle might be more effective than the TCA cycle in producing malate and oxaloacetate replenishment during lipid biosynthesis.

In addition to the GOX and TCA cycle flux ratios, Wang et al. (2019) observed a relatively higher flux ratio of the PP pathway in MT-overexpressing strains, which suggest that the PP pathway possibly plays a significant role in supplying NADPH for fatty acid synthesis in *M. circinelloides*. However, a lack of significant difference in the ME flux between the mutant strains and parental control suggested that ME is not a limiting factor for fatty acid synthesis in *M. circinelloides*. This observation agrees with earlier findings by Wynn and Ratledge (1997), who investigated the role of ME on lipid accumulation in *Aspergillus nidulans*.

Chang et al. (2019) explored the role of AMPD expression on lipid accumulation in *Mortierella alpina* under fermentation conditions by utilizing recombinant homologous AMPD-overexpressing *M. alpina* strains. It was observed that the overexpression of homologous AMPD in *M. alpina* favored lipid synthesis above cell growth. In addition, a concomitant reduction (by 40–60%) in intracellular adenosine monophosphate (AMP) and a 1.9 to 2.7-fold increase in citrate content compared with the control was observed at the early stages of fermentation. The increase in citrate suggests the provision of more precursors for fatty acid synthesis, while the decrease in AMP level indicates a metabolic reprogramming, which involves the channeling of more carbon toward lipid synthesis pathways.

β-oxidation is the main catabolic pathway of fatty acid degradation. Because lipid accumulation is the metabolic balance between lipidogenesis and degradation, the activity of β-oxidases competes with the accumulation of lipids. The β-oxidation pathway consists of a four-step cycle that eliminates two carbons (an acetyl-CoA molecule) from the acyl-CoA chain during each cycle (Courchesne et al., 2009). Thus, by blocking this lipid biosynthesis -competing pathway and by increasing the availability of lipid precursors, such as acetyl-CoA, lipid accumulation can be significantly enhanced in oleaginous filamentous fungi (Ledesma-Amaro et al., 2015; Díaz-Fernández et al., 2017). To this end, Ledesma-Amaro et al. (2015) constructed fatty-acyl coenzyme A oxidase (encoded by *POX1*) and acyl-CoA oxidase (encoded by *FOX1*) knock-out mutants of *Ashbya gossypii*, using *A. gossypii* strains which had been integrated with heterologous *ACL1 and ACL2* genes (from *Yarrowia lipolytica*) for the expression of ATP-citrate lyase activity (the wild-type *A. gossypii* strain lack ACL). By blocking the first step of β -oxidation through the knock-out of *POX1*, the authors observed an increase in lipid accumulation (from ∼15% in the wild type to 70% in the mutant) in media containing 1% glucose and 2% oleic acid after 7 days of cultivation. Thus, demonstrating that blockage of lipid accumulation competing pathways in oleaginous filamentous fungi is a viable approach for enhancing the yields of SCO.

Other lipid biosynthetic-related enzymes which determine the degree of saturation and accumulation of specific fatty acids of interest are desaturases, such as those that catalyze the conversion of oleic acid to linoleic acid or gamma-linoleic acid (GLA, 18:3, n−6). Toward enhancement of GLA production in *M. circinelloides*, Zhang et al. (2017) constructed recombinant strains with homologous overexpression of Δ6 desaturases. It was observed that this increased the GLA proportion by 38% of the total fatty acids and the yield by 33%, thereby demonstrating that the Δ6 desaturase-overexpressing *M. circinelloides* strain can potentially be used for enhanced microbial GLA production. In turn, GLA can be further converted to dihomo-gamma linolenic acid (DGLA, 20:3, n−6), an elongated product of GLA catalyzed by the enzyme Δ6 elongase (D6E) or γ-linolenic acid elongase (Yazawa et al., 2007). In a study by Khan et al. (2019b), a D6E overexpression strain of *M. circinelloides* (generated from *M. circinelloides* CBS277.49) showed an increase of up to 5.75% DLGA. Thus, this demonstrated that the overexpression of D6E in *M. circinelloides* through genetic engineering can be used for enhanced production of DLGA, an important nutritional and medical fatty acid.

## Consolidating Processing Units for Efficient Production of SCOs

### On-Site Enzyme Production and Hydrolysis

Biodiesel production typically involves multiple processing steps that are energy-intensive and contribute to high operating costs (Chopra et al., 2016). Filamentous fungi possess several traits that can be manipulated in the unit process consolidation. Some of the most attractive traits of filamentous fungi include their high growth rates and the ability to secrete an array of hydrolytic enzymes. Such traits confer filamentous fungi’s unique ability to colonize and degrade various substrates (Jun et al., 2011; Zhao et al., 2017). Microbial lipid production from low-cost lignocellulosic and agro-waste material is a promising avenue (Jin et al., 2015), however, these substrates are recalcitrant, and require a pretreatment step (e.g., steam explosion) before they can be hydrolyzed and fermented. During this process, various inhibitory by-products such as organic acids, phenolic compounds and sugar degradation products are generated, which may negatively affect microbial growth (Yang et al., 2011; Zhao et al., 2017; Valdés et al., 2020). The advantage of using filamentous fungi is that they exhibit higher tolerance to these inhibitory by-products (Abghari and Chen, 2014; Zhao et al., 2017).

Enzymatic hydrolysis is another significant cost factor in biofuel processes accounting for about 15.7% of the total cellulosic ethanol production cost (Yang et al., 2010; Fang et al., 2016). On-site production of enzymes by filamentous fungi has the potential to drive down biomass to SCO production (Ahamed and Vermette, 2008; Jun et al., 2011; Fang et al., 2016). Some of the advantages of on-site enzyme production include using low-cost substrates, eliminating the cost of separation, concentration, storage, and transportation (Fang et al., 2016; Zhao et al., 2017). Furthermore, on-site enzyme production by filamentous fungi is stimulated by the lignocellulosic substrate to be hydrolyzed, resulting in increased enzymatic hydrolysis specificity for that particular substrate (Fang et al., 2016).

Multi-stage enzymatic hydrolysis using mixed fungal cultures of *Trichoderma reesei* and *Aspergillus niger* has been reported in SCO production as an excellent approach to achieve an optimum on-site production of cellulases (Yang et al., 2010; Zhao et al., 2017). Various cellulases produced by filamentous fungi can degrade the substrate synergistically. Zhao et al. (2017) showed that the synergistic interaction of enzymes produced by a mixed culture of *T. reesei* and *A. niger* had a cellulose hydrolysis potential, which was two or three times higher than that seen in monocultures. Notably, *T. reesei* produced a full set of cellulases in these mixed cultures, but its β-glucosidase activity was lower than required to achieve efficient hydrolysis. The complementary partner, *A. niger*, produced a strong activity of β-glucosidase, which allowed for efficient hydrolysis (Jun et al., 2011; Zhao et al., 2017). Mixed culture fermentations are preferred over monoculture fermentations because they provide better substrate utilization, increased productivity, resistance to contaminants, and better adaptability to changing conditions (Ahamed and Vermette, 2008).

Yang et al. (2010) explored a three-stage hydrolysis (6, 6, and 12 h) configuration to shorten the hydrolysis time. They found that in the three-stage hydrolysis, the yields produced from steam-exploded corn stover reached 70.2% in 24 h, whereas single-stage hydrolysis required 72 h to achieve the same yield. Further to this, Yang et al. (2011) extended the evaluation of the three-stage hydrolysis, and observed that the hydrolysis of steam-exploded corn stover increased by approximately 37% compared to single-stage hydrolysis, reaching 85.1% in 30 h. To demonstrate the on-site three-stage hydrolysis with the production of SCOs, Fang et al. (2016) evaluated a mixed culture system composed of *T. reesei* and *A. niger* during the conversion of corn stover to SCOs by *Mortierella isabellina*. In this study, a three-stage enzymatic hydrolysis process produced the highest lipid content of 57.34%. In a similar study, Zhao et al. (2017) looked at the potential of *Trichosporon cutaneum* in producing SCO for biodiesel production, when grown on steam pretreated corn stover. For this, they evaluated various mixed cultures of *T. reesei* and *A. niger* as on-site producers of cellulose enzymes, before fermentation using *T. cutaneum*. Their results agreed with earlier studies where they found that using a mixed culture of *T. reesei* and *A. niger* resulted in enhanced on-site cellulase production (Zhao et al., 2017). Moreover, the fermentation time was reduced from 72 h to 30 h, yielding about 74% at a cellulase dosage of 20 FPIU/g (Zhao et al., 2017). These results were encouraging and showed that multi-stage enzymatic hydrolysis could improve the yield and enzymatic hydrolysis time with less cellulase usage. Another interesting observation was that the on-site production of cellulases performed well than when Celluclast, a commercial cellulase, was added together with *A. niger* monoculture. This strongly suggests that the cellulase enzymes produced on-site are highly specific for the substrate, resulting in enhanced hydrolysis (Zhao et al., 2017).

### Fermentation Strategies

Over the years, efforts in developing industrial production of fungal lipids have led to the establishment of two fermentation strategies, submerged fermentation and solid-state fermentation (Čertík et al., 2012; Ochsenreither et al., 2016).

#### Submerged Fermentation

Submerged fermentation (SmF) are widely used in large-scale microbial lipid production facilities (Liu et al., 2019) and is performed with soluble nutrients submerged in liquid and typically involves three key steps, i.e., fermentation, cell separation, oil extraction and refining (Čertík et al., 2012; Lima et al., 2019). SmF offers several advantages, including (i) better monitoring of process parameters, (ii) a homogeneous medium which allows for efficient heat and mass transfer, and (iii) the fungal inoculum remains submerged and distributed evenly, thus favoring nutrient absorption (Lima et al., 2019). Another advantage of SmF is that it facilitates product recovery through simple filtration or centrifugation.

Fungal morphology can be modulated by cultivation conditions such as pH, dissolved oxygen, and nutrient supply (Chan et al., 2018). The fungal morphologies can range from free suspended mycelia to pellets. In SmF, fungi typically assume a pellet morphology which yields a high surface area to volume ratio and high-density products from fermentation broth with better biomass recovery via filtration (Xia et al., 2011; Chan et al., 2018). In addition, pelleted growth minimizes mating and occluding of the bioreactor impellers, reduces viscosity of the fermentation broth and allows better mass transfer of nutrients, and oxygen (Chan et al., 2018). Therefore, fungal hyphal growth induced by SmF conditions provides a cost-effective downstream harvesting option instead of a highly expensive centrifugation process, which is an obstacle for the microbe to fuel production approach (Xia et al., 2011). Although fungal hyphal growth may result in cheaper harvesting of fermentation products, controlling this morphology in fermentation processes might be difficult and may cause problems with oxygen transfer in liquid media. Other limitations in SmF processing conditions include the consumption of substantial amounts of water and electricity for aeration, stirring and temperature control (Colla et al., 2016; Liu et al., 2019). These inevitably result in additional costs that exceed the value of the biodiesel produced.

#### Solid-State Fermentation

Solid-state fermentation (SSF) is a promising alternative to submerged fermentation. It is gaining increasing attention as a preferred fermentation strategy for fungal-derived microbial oils (Čertík et al., 2012). In SSF configuration, the fermentation of a solid substrate proceeds in the absence of water with moisture supporting the growth and metabolic activity of the fermenting microorganism (Lizardi-Jiménez and Hernández-Martínez, 2017). The solid medium in SSF better simulates the natural habitats of fungi, results in lower wastewater output, reduced energy requirements, easy aeration and smaller bioreactor volume (Qiao et al., 2018; Liu et al., 2019). SSF exhibits several other advantages over submerged fermentation, including resistance of the fermenting microorganisms to catabolic repression when the substrate is present in abundance and the use of low-cost agro-industrial wastes (Čertík et al., 2012; Lizardi-Jiménez and Hernández-Martínez, 2017). Since various waste substrates offer different nutrient compositions, selecting the most suitable fungal species that will transform the waste materials into high-quality fatty acids that are compatible as precursors of biodiesel and other value-added products is required (Čertík et al., 2012).

Many studies on SSF focus on process and reactor design, while there are currently limited studies detailing the physiological mechanisms underlying fungal behavior in SSF (Biesebeke et al., 2002; Cheirsilp and Kitcha, 2015). Fungi exhibit hyphal growth allowing it to penetrate and colonize the solid substrate in SSF effectively. Fungal mycelia cover the surface of the substrate with the assistance of its enzymes, which helps the fungus to penetrate and colonize the substrate (Biesebeke et al., 2002). The close association of fungi with the moist substrate allows them to utilize the bound water of their substrate and their growth is not limited by the lack of free water in an SSF setup (Cheirsilp and Kitcha, 2015). Limited water content in SSF minimizes the risk of bacterial contamination in the process (Čertík et al., 2012).

However, fungal hyphal growth may also lead to gradients in substrate concentration, enzymes, water content and temperature, and the presence of substrate-air interface (Biesebeke et al., 2002). Besides, limited water content results in heat removal problems. This can be effectively addressed when there is a better understanding of oxygen consumption by fungi in SSF conditions. Uptake rates of oxygen give a better reflection of the metabolic activities related to heat production. It is well known that heat production is proportional to the respiration rate and controlling temperature to an optimum level is a difficult but essential part of SSF (Biesebeke et al., 2002). Biesebeke et al. (2002) measured the respiration rate of *Aspergillus oryzae* cultivated in wheat kernels at varying temperatures. They established that the optimum temperature for fungal respiration was between 30 and 35°C. Furthermore, they established which factor contributed to the cooling problem between the dense mycelium on the surface and the abundant aerial mycelium. The densely packed mycelium layers limited oxygen diffusion, while less diffusion limitation was observed in the aerial mycelium. This observation suggested that cooling problems in fungal SSF might be solved by selecting fungi that do not produce densely packed mycelium.

#### Fermentation Process Integration

Process consolidation strategies that remove single operational units are recognized as the future of microbial lipid production at industrial scale. Qiao et al. (2018) reported that *M. circinelloides* can produce cellulases and convert lignocellulosic material to yield high lipid content without prior pretreatment process in SSF. The authors noted that the accumulation of lipids in the 6 days of fermentation was coupled with an increase in cellulase activity. This suggested that the conversion of lignocellulosic material to lipids directly depends on enzyme production and hydrolysis of complex substrate to fermentable sugars with high cellulase activity resulting in enhanced lipid production (Qiao et al., 2018).

Previous studies have shown that an integrated onsite enzyme production and fermentation of substrate to microbial lipids can be achieved by sequential combination of both SSF and SmF (Cunha et al., 2012; Melikoglu and Webb, 2013). Cunha et al. (2012) used a combined fermentation strategy system where they utilized SSF to stimulate the production of cellulases for the hydrolysis of cellulose. The cellulose hydrolyzate was subsequently fermented using SmF. Melikoglu and Webb (2013) also evaluated the potential of a combined fermentation system by first producing hydrolytic enzymes from waste bread, through a SSF process and subsequently shifting to SmF to produce value-added chemicals. Recently, Gmoser et al. (2019) reported a two-step fermentation strategy by cultivating *Neurospora intermedia* in thin stillage to initiate fungal biomass, enzyme production and hydrolysis in a SmF process, before switching to SSF (Gmoser et al., 2019).

## Considerations for SCO Recovery Methods

**FIGURE 3:**
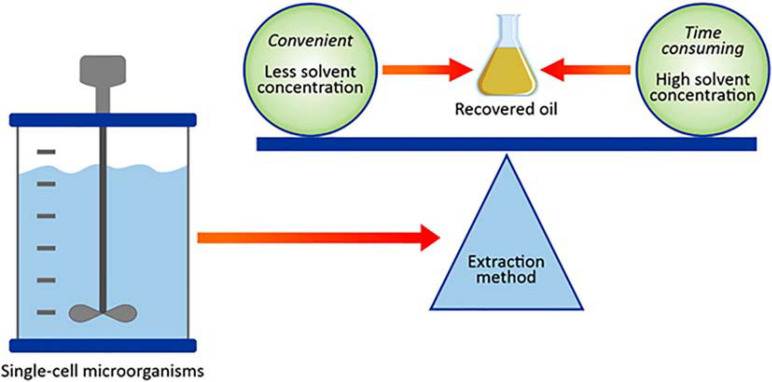
Considerations for single-cell oil recovery methods.

Single-cell oil recovery is aimed at efficiently separating the desired oils from other cellular components, such as proteins and polysaccharides. For efficient oil extraction, time of extraction, associated costs, the characteristics of the desired lipids as well as of the microorganisms have to be considered (Figure 3; Mustafa and Turner, 2011). In addition, when selecting the extraction method, the solvents to be used for extraction must also be considered. The choice of solvents is dependent on the polarity of lipids to be extracted. For instance, lipids such as TAGs, due to their neutrality, can be extracted using non-polar solvents such as hexane, while polar solvents such as alcohols are used for phospholipid extraction. However, solvent mixtures have been shown to enhance extraction efficiency (Byreddy et al., 2015). This is attributed to a polar solvents’ ability to release lipids from protein-lipid complexes and subsequent dissolution in the non-polar solvent.

Conventionally, the extraction of lipids from microbes is conducted using Soxhlet or Folch techniques (Neto et al., 2019). These techniques firstly involve biomass recovery from the broth through centrifugation and subsequent drying before solvent extraction. Oil recovery from dry biomass is considered to be efficient, however, there are economical and safety shortcomings involved. Drying of biomass involves extensive energy use and may also lead to substantial compound degradation due to high reaction temperatures (Xu et al., 2011; Neto et al., 2019). Soxhlet extraction requires extended durations of extraction and high solvent quantities (Somashekar et al., 2001). However, the method has been improved for the enhancement of extraction capabilities. For instance, a microwave-assisted Soxhlet extraction has been more efficient by reducing extraction time and solvent consumption than Soxhlet extraction alone (García-Ayuso et al., 2000). Therefore, the cost is also considerably reduced. Even though Soxhlet and Folch extraction methods have been used for SCO recovery, they have been found to be not as efficient in extracting single oil classes compared to total lipid extraction (McNichol et al., 2012). Therefore, both methods require modifications when the focus is to recover oils from single-cell microorganisms (Ochsenreither et al., 2016).

Cell disruption methods have the potential to enhance oil recovery yields (Koubaa et al., 2020). The most investigated technologies include bead milling, high-pressure homogenization (HPH), ultrasound- and microwave-assisted extraction (UAE and MAE, respectively). The degree of cell disruption is dependent on various factors such as the type of microorganism tested, the concentration of the microorganism in the growth medium, as well as cell wall composition and structure. Among cell disruption technologies for recovering oil, bead milling is considered the most efficient method (Koubaa et al., 2020). It involves the use of beads for grinding biomass against the solid surfaces during violent agitation (Dong et al., 2016). High-pressure homogenization involves passing a suspension through a high-pressure valve. Although the mechanisms responsible for cell disruption when using HPH are still vague, it has been hypothesized that the high-velocity effects of the fluid passing through the valve as well as differential pressure between the valve cause cell wall damage and subsequent release of the intracellular compounds (Samarasinghe et al., 2012). Zhou et al. (2013) investigated UAE of SCOs from *Mortierella isabellina*. The yield of lipids recovered was approximately 109.88 mg/g, which was 1.35-fold the yield of the acid-heating method, suggesting that UAE might be a cost-effective and sustainable method for SCO extraction. Thus, UAE can have the potential for large-scale oil extraction technology from microorganisms (Zhou et al., 2013).

Microwave application for the disruption of microbial cells has also been extensively studied (Woo et al., 2000; Campanha et al., 2007; Dong et al., 2016). The major mechanism involved in the microwave extraction is heating due to the rise in pressure and temperature. The phenomena are responsible for damaging cell walls, releasing intracellular compounds. Zhou et al. (2018) studied the efficiency of extraction and the quality of SCO from *M. isabellina* using ultrasound in combination with the microwave extraction method. It was observed that the combined extraction method improved the quality and yield of SCO. This can potentially translate to cost reduction due to reduced time and energy usage for extraction.

Nanoparticles can be used to maximize oil recovery (Dong et al., 2016; Negin et al., 2016). However, the application of nanoparticles for SCO recovery is still in its infancy. Depending on the process conditions during extraction, various nanoparticles perform differently due to their characteristics such as particle size and surface area to volume ratio, leading to different levels of lipid recovery. According to Bjørnar (2012), the efficacy of these nanoparticles is due to their small size, making it possible for the nanoparticles to penetrate through pore spaces where conventional recovery techniques cannot, thus, resulting in higher recovery. Therefore, more research devoted to nanoparticle behavior during SCO recovery is pertinent for developing fast and convenient extraction methods. Furthermore, research work on the use of “green” extraction solvents such as terpenes in place of the currently used solvents (e.g., hexane, chloroform, etc.) is necessary to promote eco-friendly oil extraction methods.

The advantages and disadvantages of different cell disruption techniques for oil recovery are summarized in Table 5.

**TABLE 5:** Advantages and disadvantages of single-cell disruption methods for SCO recovery.

**SCO recovery method**	**Advantages**	**Disadvantages**
Bead milling	Efficient and less complicated	Not effective for bacteria
Ultrasonic	Allows for continuous runs	Heat generation
High pressure homogenization	Currently in use in several industries for various applications and is effective	Not suitable for filamentous fungi
Microwave	Rapid and cost-effective	Heat generation
Filamentous fungal pellet	Mainly effective in isolating fungal cells	Limited application

The selection of a suitable cell disruption technology for oil recovery and cost-benefit analysis toward the final product requires more research attention. Presently, most studies on energy consumption of conventional methods for different processes have primarily focused on time and temperature reduction (Chemat et al., 2017; Postma et al., 2017; Roohinejad et al., 2018; Gaudino et al., 2019). The lack of information on energy usage makes it difficult to estimate overall costs for scaling-up the process.

## Toward a Biorefinery Concept

Although it has been established that several fungal species can accumulate SCOs, which may be harvested and utilized for value-added product generation, high fermentation costs have often restricted industrial application of such species (Kothri et al., 2020; Patel et al., 2020). However, the combination of fungal SCO production with several other biotechnological applications, in a biorefinery concept, could help minimize costs (Kothri et al., 2020). A biorefinery refers to a facility that utilizes various technologies to enable complete processing solutions, thereby resulting in an array of bioproducts with minimal waste generation. Such bioproducts include biomaterials (e.g., pulps, fibers), biofuels (e.g., biogas, biodiesel, bioethanol), and various bioactive compounds (e.g., enzymes, anti-bacterial agents), which collectively contribute to improving the value derived from the feedstock in use (Yousuf et al., 2018). Filamentous fungi have received attention as promising candidates for adoption in biorefineries, due to their ability to synthesize a multitude of macromolecules such as SCOs, enzymes, structural polymers, and compounds with pharmaceutical and nutraceutical applications (Reis et al., 2019).

In comparison to algal biorefineries, minimal studies have been conducted to test the feasibility of oleaginous fungi for biodiesel production in biorefineries, and in most instances, such studies were only laboratory-based. One such study, conducted by Kamat et al. (2013), tested two fungal species, isolated from a mangrove for their ability to produce lipids, biodiesel, xylitol and xylanase from sugarcane bagasse. The synthesized lipids were well within the international standards for use as a biodiesel feedstock and the xylanase was reported to have the potential for application in the seafood industry. Carvalho et al. (2019) tested *M. circinelloides* for its ability to produce SCOs from sugarcane bagasse hydrolyzate. They found that *M. circinelloides* was able to synthesize fuel-grade ethyl esters and PUFAs for potential use in the nutraceutical industry. Moreover, they found that the fungal biomass presented with lipolytic activity and the fungal lipids contained carotenoids indicating that *M. circinelloides* has the potential to generate several high-value products. While research on fungal biorefineries is less prominent, there is a substantial motivation for more research to be channeled in this direction as the extracted biomass may still be utilized for bioethanol, biomethane or biohydrogen production. For example, Bagy et al. (2014) tested a two-stage system where six strains of oleaginous fungi were cultivated in sugarcane molasses for biodiesel production (first stage). The spent fungal culture medium was then utilized for biohydrogen production (second stage) by *Clostridium acetobutylicum* ATCC 824 to improve the process’s economic feasibility.

Biohydrogen has numerous advantages over other biofuels such as biogas and bioethanol. The main advantages are that biohydrogen has elevated energy density and the significant emission from its utilization in a hydrogen fuel cell, is solely water. However, its commercial production is impeded due to elevated production costs. Biohydrogen is produced during dark fermentation. The by-product of the production process is a liquid waste (HPLW), which can be utilized as a feedstock for lipid production (Sarma et al., 2015). Hence, the integration of biohydrogen and lipid generation in a biorefinery may offset the elevated costs associated with sole production of biohydrogen. One such concept was proposed by Béligon et al. (2018) where they tested the feasibility of an immersed membrane bioreactor containing individual reactor compartments for biohydrogen and SCO production. In their study, the first bioreactor compartment was structured to promote dark fermentation of lignocellulosic matter to biohydrogen and volatile fatty acids (VFA). The second compartment comprised oleaginous yeast, *Cryptococcus curvatus*, which has the ability to convert the VFA-rich effluent from the first compartment to SCOs. A similar concept could be used when integrating oleaginous filamentous fungi into biorefineries.

**FIGURE 4:**
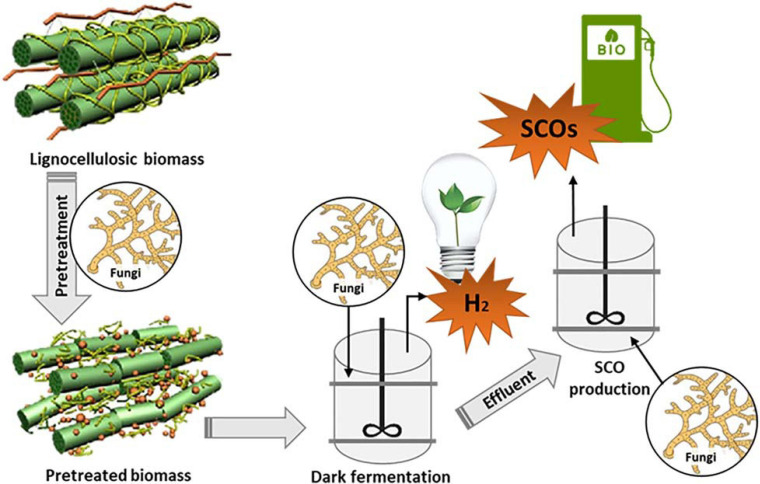
Combining biohydrogen (H_2_) and single cell oil (SCO) production to increase the efficiency and economic feasibility of the process.

It is therefore proposed that more studies be aimed at testing the economic and technical feasibility of integrating biohydrogen and SCO production, particularly from oleaginous filamentous fungi (Figure 4). Like with the study by Béligon et al. (2018), it is suggested that low-cost lignocellulosic material be utilized as the feedstock for the proposed biorefinery. This will substantially reduce input costs and improve economic viability. Filamentous fungi may find applications in all stages of the biorefinery process due to their ability to produce an array of extracellular enzymes (Troiano et al., 2020). Thus, they may be utilized for the biological pretreatment of the lignocellulosic substrate prior to dark fermentation, which would enable the breakdown of the lignocellulosic structure. This can improve the accessibility of cellulose and hemicellulose, and maximize biohydrogen production (Liu and Qu, 2019). Facultative or obligate anaerobic fungal strains may also be utilized as an inoculum during the biohydrogen production process as proven in a study by Nkemka et al. (2015), where bioaugmentation with the fungal strain, *Piromyces rhizinflata*, resulted in increased initial biohydrogen and biomethane production rates in a two-stage reactor. Increased initial biogas production rates will in turn, reduce the required digestion time, which will reduce the cost implications of the process (Nkemka et al., 2015). Oleaginous fungal species may then be utilized to convert the effluent, after biohydrogen production, into SCOs. A combination of low and high-value SCOs will improve the cost-effectiveness of the process. The upgrading of the final effluent after extraction of the lipid-rich cells will also be beneficial in improving economic viability (Jin et al., 2015). A cost-intensive aspect of SCO production is the harvesting of biomass prior to lipid extraction. The use of oleaginous fungi for SCO production is beneficial since fungal mycelia’s entanglement decreases medium viscosity and eases cell harvesting (Reis et al., 2019). A recent study by Yang L. et al. (2019) showed that the co-cultivation of oleaginous algae and fungi aided in harvesting the resulting lipids and improved lipid quality in comparison to mono-cultivation. This could be another strategy that can be adopted in a SCO biorefinery to improve the quality of SCOs with downstream economic implications.

Overall, several factors influence the success of an SCO biorefinery. Such factors include strain selection, choice of feedstock, method of substrate pre-treatment, the configuration of the production process, method of cell harvesting and lipid recovery as well as the number and type of co-products generated. Various strategies can be put into place to address the factors mentioned above, such as strain genetic modification, optimization of culture conditions and technological improvements of process configurations (Jin et al., 2015). All in all, such strategies are aimed at overcoming cost implications, which is the major bottleneck in SCO production.

## Conclusion

Single-cell oils have the potential to provide a sustainable alternative for obtaining PUFA-rich lipids. Hence, researchers have been focusing on increasing the yield of SCO for its commercial realization. For SCOs to be considered commercially feasible, the production process must be conducted at ideal conditions for optimal efficiency and minimum costs. This could be achievable by making use of relatively inexpensive substrates such as agro-industrial waste. Despite, low-cost starting material, the process of SCO production in combination with downstream processing still makes it cost-intensive. Hence, the paper also discussed possibilities of reducing costs by utilizing SCO co-production with other prospective metabolic products such as biohydrogen in a biorefinery approach to balance production cost, thereby circumventing overall cost challenges. Aside from improving economic feasibility, a biorefinery approach will also help achieve several sustainable development goals and promote a sustainable circular economy. In addition, the quest for developing tolerant, robust and high yielding microbial strains for efficient oil production would also play a vital role for a sustainable holistic approach.

## Author Contributions

SM conceptualized and prepared the initial outline of the manuscript. SM, OE, AR, BN, and PS wrote and edited the first draft of the manuscript. OH and CP were responsible for critical review, literature gathering, and overall improvement of the manuscript. All authors read and approved the final draft of the manuscript.

## Conflict of Interest

The authors declare that the research was conducted in the absence of any commercial or financial relationships that could be construed as a potential conflict of interest.
